# Diversity and substrate-specificity of green algae and other micro-eukaryotes colonizing amphibian clutches in Germany, revealed by DNA metabarcoding

**DOI:** 10.1007/s00114-021-01734-0

**Published:** 2021-06-28

**Authors:** Sten Anslan, Maria Sachs, Lois Rancilhac, Henner Brinkmann, Jörn Petersen, Sven Künzel, Anja Schwarz, Hartmut Arndt, Ryan Kerney, Miguel Vences

**Affiliations:** 1 Zoological Institute, Technische Universität Braunschweig, Braunschweig, Germany; 2 Institute of Zoology, University of Cologne, Zülpicherstr. 47b, 50674 Köln, Germany; 3 Leibniz Institute DSMZ - German Collection of Microorganisms and Cell Cultures, Inhoffenstraße 7B, 38124 Braunschweig, Germany; 4 Department of Evolutionary Genetics, Max Planck Institute for Evolutionary Biology, 24306 Plön, Germany; 5 Institute of Geosystems and Bioindication, Technische Universität Braunschweig, Braunschweig, Germany; 6 Department of Biology, Gettysburg College, Gettysburg, PA USA

**Keywords:** DNA metabarcoding, Symbiosis, *Oophila*, *Rana dalmatina*, Amphibians, Algae

## Abstract

Amphibian clutches are colonized by diverse but poorly studied communities of micro-organisms. One of the most noted ones is the unicellular green alga, *Oophila amblystomatis*, but the occurrence and role of other micro-organisms in the capsular chamber surrounding amphibian clutches have remained largely unstudied. Here, we undertook a multi-marker DNA metabarcoding study to characterize the community of algae and other micro-eukaryotes associated with agile frog (*Rana dalmatina*) clutches. Samplings were performed at three small ponds in Germany, from four substrates: water, sediment, tree leaves from the bottom of the pond, and *R. dalmatina* clutches. Sampling substrate strongly determined the community compositions of algae and other micro-eukaryotes. Therefore, as expected, the frog clutch-associated communities formed clearly distinct clusters. Clutch-associated communities in our study were structured by a plethora of not only green algae, but also diatoms and other ochrophytes. The most abundant operational taxonomic units (OTUs) in clutch samples were taxa from *Chlamydomonas*, *Oophila*, but also from *Nitzschia* and other ochrophytes. Sequences of *Oophila* “Clade B” were found exclusively in clutches. Based on additional phylogenetic analyses of 18S rDNA and of a matrix of 18 nuclear genes derived from transcriptomes, we confirmed in our samples the existence of two distinct clades of green algae assigned to *Oophila* in past studies. We hypothesize that “Clade B” algae correspond to the true *Oophila*, whereas “Clade A” algae are a series of *Chlorococcum* species that, along with other green algae, ochrophytes and protists, colonize amphibian clutches opportunistically and are often cultured from clutch samples due to their robust growth performance. The clutch-associated communities were subject to filtering by sampling location, suggesting that the taxa colonizing amphibian clutches can drastically differ depending on environmental conditions.

**Supplementary Information:**

The online version contains supplementary material available at 10.1007/s00114-021-01734-0.

## Introduction

Amphibians are characterized by a striking diversity of reproductive modes (Haddad and Prado 2005; Salthe and Duellman 1973) that also is reflected in the diversity of eggs and clutches they deposit (Altig and McDiarmid 2007). Amphibian eggs are typically surrounded by a vitelline membrane and several egg capsule layers suspended in a jelly matrix consisting of mucopolysaccharides secreted by the oviduct (Salthe 1963). In many cases, single eggs with their surrounding jelly layers are combined into complex clutches, strings, or sacs. Studies on water molds (Johnson et al. 2008; Petrisko et al. 2008), bacterial (Hughey et al. 2017), and micro-eukaryotic organisms (Jurga et al. 2020) suggest that amphibian eggs are colonized by diverse but poorly studied communities of prokaryotic and eukaryotic organisms, some of which may be specifically adapted to this micro-ecosystem.

One of the most prominent organisms associated with amphibian eggs is the unicellular green alga, *Oophila amblystomatis.* This alga is characterized by a close and mutualistic association with amphibian eggs and embryos from multiple species. The singular interactions of *Oophila* with its amphibian hosts, in particular with North American salamanders of the genus *Ambystoma*, were noted as early as in the late nineteenth century (Orr 1888). In these salamanders, the embryo exits the vitelline membrane during neurulation (see Altig and McDiarmid 2007; Salthe 1963 for details of egg capsule structure), and a bloom of *O*. *amblystomatis* proliferates outside the embryonic blastopore. *Oophila* within the capsular chamber provide an increase in the partial pressure of oxygen during the day, potentially remove nitrogenous waste, and have been reported to transfer photosynthate to the amphibian embryos (Bachmann et al. 1986; Goff and Stein 1978; Graham et al. 2013; Kerney 2011; Pinder and Friet 1994), which may lead to acceleration of embryonal development, larger sized embryos, increased viability and hatching success (Gilbert 1942, 1944). However, other studies have reported no measurable exchange of photosynthate from algae to amphibian embryos (Burns et al. 2020). Additional effects by the algae, such as a reduction of micro-organisms that are potentially harmful to the host, have been hypothesized (Kim et al. 2014) but not yet tested. Potential benefits for the algae are less studied. It is suggested that the capsular chamber of amphibian eggs provides a protective environment, acts as an insulator, and thereby offers higher temperatures than the pond water (Beattie 1980). Additionally, it has been argued that the host embryos may provide nitrogenous compounds to algae (Goff and Stein 1978), yet this hypothesis remains controversial (Bianchini et al. 2012; Small et al. 2014). The close symbiotic association of *Oophila* with its amphibian hosts even leads to algal cells invading host embryonic tissues and cells in the spotted salamander (*Ambystoma maculatum*), and thus constitutes a unique example of endosymbiosis in vertebrates (Kerney et al. 2011, 2019).

Despite the considerable amount of research carried out on *Oophila*, numerous aspects of this mutualistic amphibian-algae system remain insufficiently studied. These include even fundamental questions such as the taxonomic identity and global distribution of these amphibian-associated algae, host specificity, and ecology of the algae outside of the amphibian breeding season. Molecular work based on algal cultures isolated from amphibian clutches (Kim et al. 2014; Muto et al. 2017) and DNA metabarcoding (Jurga et al. 2020) found almost all DNA sequences belonging to one clade of green algae. This clade had a somewhat isolated (phylogenetic) position within Chlamydomonadales, and besides, the clutch-associated algae contained only three isolates of free-living algae. Isolates of this clade have been consistently considered as *O. amblystomatis*, which is regarded as the numerically most abundant alga in *A*. *maculatum* (Jurga et al. 2020) and *A*. *gracile* (Kerney et al. 2019; Marco and Blaustein 2000) egg capsule chambers. These *O. amblystomatis* isolates have been used in studies of gene expression (Burns et al. 2017; Kerney et al. 2019), carbon fixation (Burns et al. 2020), and host-symbiont fidelity (Kerney et al. 2019). As an exception, a few isolates from *A*. *maculatum* egg masses, along with environmental samples, were assigned to *Chlamydomonas gloeophila*; however, *C*. *gloeophila* was suggested to represent low abundance green algae occurring in these egg masses that outcompeted *Oophila* under agar media growth conditions in culture (Kim et al. 2014).

A recent study by Nema et al. (2019), in contrast, found algae isolated from amphibian clutches to be phylogenetically diverse. They considered their new isolates from near the type locality as *Oophila* “Clade A”, purportedly representing the true *O. amblystomatis*, and referred to the previously studied algae as *Oophila* “Clade B”. This interpretation is based on molecular sampling done in another unpublished study (Lewis and Landberg 2014) and the unpublished naming of *O. amblystomatis* by Lambert (Printz 1927) from samples collected outside of Boston, MA. Because “Clade A” sequences are closely related to species of *Chlorococcum*, Correia et al. (2020), relying on the taxonomic conclusion of Nema et al. (2019) suggested to re-name the species as *Chlorococcum amblystomatis*. While a conclusive taxonomic decision on the identity of *Oophila* will require more in-depth study of the (preserved) material used for the original description of the taxon, some hints on the conundrum can be also obtained by better understanding the ecology of these algae. In particular, by addressing the following topics, it might be possible to distinguish between actual amphibian clutch symbionts (very likely corresponding to those algae originally described as *Oophila*) and opportunistic generalists that occur in low abundances in this habitat but may be more easily cultured: (i) assessing which alga is numerically most abundant in amphibian clutches, (ii) under which ecological conditions and in which developmental stage different algae and other micro-organisms may opportunistically invade these clutches, and (iii) whether specific culturing conditions may favor low-abundance algae relative to the strains of *Oophila* “Clade B”.

Besides green algae, the occurrence and role of other micro-organisms in the capsular chamber surrounding amphibian eggs, and in amphibian clutches in general, also remain mostly unstudied. Bacterial communities associated with frog clutches are similar to those on the skin of adult frogs (Hughey et al. 2017). As the cutaneous microbiome in amphibians provides an important defense line against pathogenic fungi (Bletz et al. 2013), it may be speculated that clutch-associated bacterial microbiome may represent an extended component of the embryo’s pathogen defense. Recent studies have also shown that one of the most important amphibian pathogens, the chytrid fungus *Batrachochytrium dendrobatidis* that has caused dramatic amphibian declines globally (Fisher and Garner 2020) is mitigated by the presence of micro-eukaryotes predating on its zoospores (Schmeller et al. 2014). Protozoan micro-predators (Yassin and El-Said 2011) and probiotic bacteria (Chauhan and Singh 2019) have also proven effective in reducing the growth of *Saprolegnia* water molds that are known to cause extended mortality of amphibian eggs (Blaustein et al. 1994; Gomez-Mestre et al. 2006). Recently, Jurga et al. (2020) used a DNA metabarcoding approach to demonstrate that fluid from the capsular chambers of *A. maculatum* clutches contained communities of micro-eukaryotes that represented a subset of the aquatic taxa present in free water at the respective sampling sites. Besides *Oophila*, also cercozoan protists and chytrid fungi were sometimes abundant. Given the diversity of the jelly layer structure in different amphibians with differences sometimes detected even in the same species (Beattie 1980) and the variation of microbes depending on habitat properties (Bock et al. 2020; Montiel et al. 2019), substantial differences can be expected in the communities of organisms inhabiting the amphibian clutch micro-environment.

Driven by our own field observations in Germany suggesting regular occurrence of micro-algae in the capsular egg chambers in clutches of agile frogs, *Rana dalmatina*, we undertook a multi-marker DNA metabarcoding study to better characterize the community of algae and other micro-eukaryotes associated with these clutches. To assess the occurrence and taxonomic identity of micro-organisms associated with *R. dalmatina* clutches, we pursued three main research questions: (i) Which species and strains of algae occur in clutches of *R. dalmatina* (in Germany) and how are these related to the strains identified from North America and Japan? (ii) Do *Oophila* and other amphibian clutch-associated algae occur also elsewhere in the pond environment? (iii) Lastly, what are the evolutionary relationships of algae isolated from amphibian clutches to other Chlorophyta? This final question required combining sequences derived from transcriptomes and DNA metabarcoding, along with the sequences from previous studies, for taxonomic and phylogenetic analyses.

## Methods

### Sampling

**Fig. 1:**
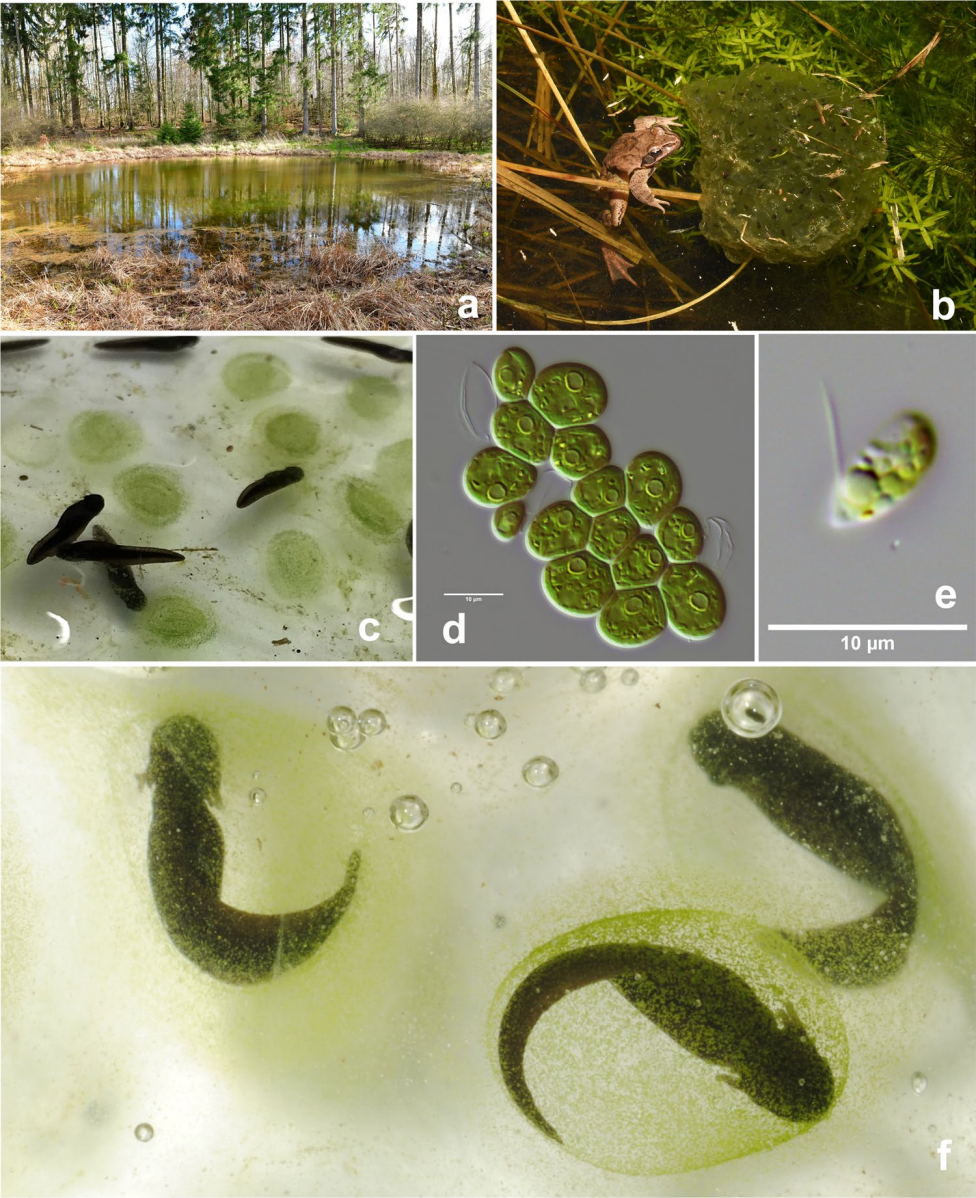
Clutches and eggs of *Rana dalmatina* from sampled ponds with unicellular green algae in the capsular chamber. (**a**) Pond “Lelm1” in early spring after the main spawning event. (**b**) Male *Rana dalmatina* with a green-colored clutch due to development of green algae within this egg mass. (**c**, **f**) Close-up images of early tadpole stages and remains of egg clutches with green algae in the inner egg capsule. Non-motile zygotes (**d**) and a flagellated zoospore (**e**) of these algae

Sampling was performed at three small ponds in Germany, all located in the Elm region near Braunschweig, here named Lelm1, Lelm2, and Dahlum (Online Resource 1, Table S1; Fig. 1a). Samples were collected from four types of substrates: (1) water, (2) sediment, (3) tree leaves from the bottom of the pond, and (4) *R. dalmatina* clutches (Fig. 1b). Water samples were collected via an algal net (0.25 µm mesh) by filtering and concentrating water into the 100 ml collection bottle (scooping the algal net in the center of the pond eight times across ca. 3 m). Sediment samples were collected from six random locations from the bottom of the pond by collecting a total of ca. 450 g from the top 2 cm layer. Nine tree leaves (elm or oak) were collected per pond at random locations into the 50 ml sterile tubes. When the frog clutches emerged at the ponds at random location, jelly samples from approximately 30 eggs were collected for *R. dalmatina* by placing the samples into a sterile 50 ml tube, after photographing the clutch to determine developmental stage. The first sampling of clutches was performed on the 25^th^ of March 2019 and the last on the 18^th^ of April 2019 (Lelm 1 and Lelm2) and 11^th^ of April 2019 (in Dahlum sampling site, i.e., no clutches in Dahlum on 18^th^ of April 2019). Total number of collected samples was 100; 24 for water, sediment and leaf samples and 28 for clutch samples (Online Resource 1, Table S1). All samples, except clutches, were frozen at − 20 °C (maximum of 2 h after collection) until further processing.

The algal colonization rate and embryo developmental stage were recorded for *R*. *dalmatina* clutch samples (Online Resource 1, Table S1; Fig. 1c–d). Embryo developmental stages were categorized to (i) egg to very early embryo, i.e., approximately corresponding to stages 1–14 according to the classification of Gosner (1960); (ii) early to moderately developed embryo, larval form not yet fully developed, approximate Gosner stages 15–19; (iii) hatchling with external gills but still inside egg, approximate Gosner stages 20–22; (iv) hatched embryo, approximate Gosner stages over 22. In the laboratory, clutch samples in 50 ml tubes were immediately poured onto Petri dishes, where ca. 4 ml of the clutches mass (without embryos) were transferred (with sterile 2 ml syringes) to new sterile 50 ml tubes avoiding the obvious outer ‘environmental contamination’ as much as possible. About 20 ml of RNA later solution was added on top of each 4 ml clutch sample, briefly vortexed, and stored at 4 °C. Detailed sample preparation for DNA extractions is described in Online Resource 2 (extended methods). Because only jelly samples of the clutches were collected and no larval or metamorphosed vertebrates were manipulated, ethics approval for our study was not required. Permission to handle amphibians in the study ponds was granted by the Untere Naturschutzbehörde of the Landkreis Helmstedt (16/605,105–269/14 and 16/605,105–074/21).

### Molecular analyses

All DNA extractions were performed using DNeasy PowerSoil Kit (Qiagen, Germany) following the manufacturer’s instructions. PCRs were performed using uniquely tagged primers (8 bp + 2–4 bp heterogeneity spaces; Online Resource 1, Table S2) for amplifying fragments of RuBisCO large subunit (rbcL) and eukaryotic small subunit of ribosomal RNA (18S SSU rRNA). For amplifying the rbcL region, we used the newly designed primers, rbcL-646Fcl (5′-ATG CGT TGG MGW GAY CGT TTC-3′) and rbcL-998Rcl (5′-GTT CHC CTT CWA RTT TWC CWA CWA C-3′), modified from Kelly et al. (2018; rbcL646F and rbcL998R, designed for diatoms) to amplify a wider range of photosynthetic micro-algae (targeting especially Chlamydomonadales). These new rbcL primers amplify a fragment of 333–336 bp. For amplifying 18S (V9 region), we used universal primers Euk1391f (5′-GTA CAC ACC GCC CGT C-3′) and EukBr (5′-TGA TCC TTC TGC AGG TTC ACC TAC-3′) (Amaral-Zettler et al. 2009; Stoeck et al. 2010). PCR mix, 25 µl per sample, consisted of 5 µl of Hot Start FirePol Master Mix (Solis BioDyne, Estonia), 0.5 µl forward and reverse primers (10 µM), 1 µl of template DNA, and 18 µl of nuclease-free water. PCR conditions for rbcL included initial hot-start at 95 °C for 15 min, following 35 cycles of 95 °C for 30 s, 55 °C for 45 s, 72 °C for 1 min, and final extension at 72 °C for 10 min. PCR conditions for 18S included initial hot-start at 95 °C for 15 min, following 35 cycles of 94 °C for 45 s, 57 °C for 1 min, 72 °C for 1 min 90 s, and final extension at 72 °C for 10 min. Two replicate PCRs were performed per sample. Sample replicates were pooled, and the yield of PCR products were checked via gel electrophoresis by pipetting 5 µl PCR product on 1% agarose gel. All PCR products were pooled based on their relative quantity (as observed on the gel) and purified using Favor-Prep™ Gel/PCR Purification Kit (Favorgen-Biotech Corp., Austria), following the manufacturer’s instructions. Steps of DNA extraction, PCR, and sequencing included both negative and positive controls. Negative controls included blank DNA extractions and also PCRs with no-template DNA. DNA extracts from cultured species of diatoms were used as a positive control to monitor the functionality of PCRs and sequencing. Additionally, sixteen ‘un-used tag’ control samples were used (Online Resource 1, Table S2) to account for potential ‘tag-switching’ errors (Taberlet et al. 2018). All molecular procedures were performed under a laminar flow clean bench, with 30 min UV sterilization prior to and after each step. Sequencing was performed on an Illumina MiSeq instrument using MiSeq Reagent Kit v2 (2 × 250). Illumina sequencing data sets have been deposited in the Sequence Read Archive (SRA), BioProject ID: PRJNA714784.

### Bioinformatics

Raw paired-end Illumina sequencing data were processed in the PipeCraft platform (Anslan et al. 2017), which included merging paired-end reads, quality filtering, chimera filtering, clustering, and formation of operational taxonomic units (OTUs) tables for both (rbcL and 18S) genes. Paired-end reads merging and quality filtering were processed using vsearch (Rognes et al. 2016); maximum expected error threshold of 1 (–fastq_maxee = 1) and discarding sequences with ambiguous bases (–fastq_maxns = 0). Putative chimeric reads were filtered using the uchime_denovo algorithm in vsearch (default settings). Few additional reads where a full-length primer string was detected inside the sequence (i.e., ‘multiprimer artefacts’) were discarded using the PipeCraft built-in module (‘remove primer artefacts’). Clustering of the sequences was performed using the UPARSE algorithm (Edgar 2013) with a 97% sequence similarity threshold. For taxonomy assignment, representative sequences (UPARSE centroids) for each OTU were compared against the EMBL v142 (Kanz et al. 2005) reference database using the blastn algorithm (Camacho et al. 2009). Based on the control samples and blastn results, the OTU tables were further checked and filtered to remove potential contaminants and mitigate tag-switching errors. Detailed OTU table curation is described in Online Resource 2 (extended methods).

### Statistics

Permutational Analysis of Variance (PERMANOVA, with 9999 permutations) was used for detecting the effects of substrate (water, leaves, sediments, clutches), sampling site (Lelm1, Lelm2, Dahlum), and sampling date (8 sampling dates) on the OTU community composition using PRIMER v6 (Clarke and Gorley 2006). Log-transformed Bray–Curtis as well as UniFrac distance (unweighted) OTU matrices were used for the PERMANOVA analyses. UniFrac distances were calculated using the PhyloMeasures package (Tsirogiannis and Sandel 2016) in R (v3.6.2; R-Core-Team 2019) using Maximum-likelihood based phylogenetic rbcL and 18S amplicon trees generated with RAxML (Stamatakis 2014) under the GTRGAMMA model. Because sequencing depth may affect the OTU abundance (thus, community composition patterns), sequence counts per sample were used as a covariate (Type I SS). Obvious outliers were screened with non-metric multidimensional scaling (NMDS) analyses and removed prior to PERMANOVA. Distance-based linear model (DistLM) with forward selection procedure and AICc selection criterion (using PRIMER v6) was used to detect the most important factors affecting the algal (rbcL data) and micro-eukaryotic (18S data) communities associated with frog clutch samples (9999 permutations). For identifying OTUs that are consistently present in a given substrate type (i.e., indicator OTUs), indicator species analyses were performed using the ‘indicspecies’ library (De Caceres et al. 2016) in R. Interactive visualization graphs for indicator OTUs (taxa) were generated using a Krona chart (Ondov et al. 2011). Bar plots for taxonomic distributions were generated using the ‘phyloseq’ package (McMurdie and Holmes 2013) in R. Temporal distance decay of similarity of OTU composition was explored by Mantel tests using the ‘vegan’ package (Oksanen et al. 2015) in R.

### Culturing, Sanger sequencing and RNAseq

Single *R*. *dalmatina* egg envelopes were cut open with microsurgery scissors, and algal cells were extracted with a micromanipulator (Patchman NP2, Eppendorf, Germany). The obtained algal cells were cultivated in Waris H medium (McFadden and Melkonian 1986) under standard conditions (light/dark 14:10 h at 16 °C and 5000 K provided by LED daylight strips, SunLike Linear Z 560–52, Lumitronix, Germany). For DNA extraction, the culture was centrifuged at 4000 × g for 20 min at 4 °C. The pellet was re-suspended in 700 µl Genomic Lysis Buffer using the Quick DNA prep kit (Zymo Research, USA) following the manufacturer’s protocol for cell suspensions. The complete 18S rDNA was amplified in PCR reactions using 1.5 µl genomic DNA template, 12.5 µl Red Taq Polymerase Master Mix (VWR Chemicals International, Belgium), and each 2.5 µl 18S for 5′-AAC CTG GTT GAT CCT GCC AGT-3′ and 18S-Rev 5′-TGA TCC TTC CGC AGG TTC ACC TAC-3′ primer (Medlin et al. 1988). The thermal amplification program followed Schoenle et al. (2019): initial denaturation at 98 °C for 2 min, followed by 35 cycles of 30 s at 98 °C, 45 s at 55 °C and 2 min 30 s for 72 °C, and ending with a final elongation step of 72 °C for 10 min. PCR products were purified using the PCR purification kit (Jena Bioscience, Germany) following the manufacturer’s protocol and sequenced with the corresponding amplification primers at GATC Biotech Cologne. Sequence editing and quality check were performed using the Bioedit Sequence Alignment Editor (v7.2.6; Hall 1999). The 18S sequence (isolate MVRNA93) has been deposited in Genbank (Benson et al. 2013), under accession number MW723501.

For RNA extraction, a sample of ca. 100 mg of the culture was preserved in RNAlater at − 80 °C. Details about RNA extraction are described in Online Resource 2 (extended methods). Sequencing was carried out with a High Output 2 × 150 cycle kit on an Illumina NextSeq instrument. Reads were quality-trimmed and filtered using Trimmomatic v. 0.32 (Bolger et al. 2014) with default settings (i.e., slidingwindow: 4:5, leading: 5, trailing: 5, minlength: 25). Filtered reads were used for de novo transcriptome assembly using Trinity v. 2.1.0 (Grabherr et al. 2011) following a published protocol (Haas et al. 2013). Illumina NextSeq sequencing data is deposited in the SRA, BioProject ID: PRJNA712983.

### Phylogenetics

For phylogenetic analyses, we relied mainly on sequences of the 18S rRNA gene because most previous studies focusing on *Oophila* used this gene, and numerous comparative sequences are therefore available. Analyses were performed at levels of taxonomy and sequence length to make the best use of all available data, and considering that fully combining all sequences is not feasible due to extremely different sequence lengths and sequence variation. Furthermore, we compiled multi-gene datasets from transcriptomic data containing 18 nuclear protein-coding genes. All alignments have been uploaded to Figshare (https://doi.org/10.6084/m9.figshare.14216588).

A detailed structure of the data sets (*Dataset 1–5*) for phylogenetics is described in Online Resource 2 (extended methods). Briefly:

#### Dataset 1

To understand the identity and overall placement of the various algal isolates sequenced from amphibian clutches, we assembled a data set of all 18S rRNA sequences of such algae from Genbank (Benson et al. 2013), plus a comparative selection of Chlorophyta comprising (i) all outgroups and comparative sequences used in previous publications (Correia et al. 2020; Kim et al. 2014; Muto et al. 2017; Nema et al. 2019); (ii) all sequences assigned to *Oophila* in these studies and otherwise available from Genbank; (iii) all sequences with > 98% sequence identity and > 80% sequence coverage obtained via BLAST searches in the Genbank nucleotide collection, using the most complete 18S sequences of *Oophila* “Clade A” and “Clade B” as queries, and (iv) a selection of sequences obtained via metabarcoding.

#### Dataset 2

To visualize the placement of additional Chlorophyta OTUs found by metabarcoding in different parts of the green algae tree, we used the initial full alignment from Dataset 1 but trimmed the 18S sequences to 139 bp to match the metabarcoding fragment.

#### Dataset 3

To better visualize the variation of sequences assigned to *Oophila* “Clade A” and “Clade B” relative to other related algae, we used the 18S sequences from Dataset 1 as a basis and selected those taxa that either (i) were classified as *Oophila*, (ii) were classified as *Chlorococcum* and by Dataset 1 analysis were placed in a clade with sequences classified as *Oophila*, and (iii) were nested within the *Oophila* and *Chlorococcum* clades, or were direct sister taxa to samples classified as *Oophila*.

#### Datasets 4 and 5

To obtain confirmation of algae’s placement isolated from amphibian clutches in two very distinct branches of the Chlorophyta from a genome-wide selection of markers, we used a recently published phylotranscriptomic data set across green plants (Leebens-Mack et al. 2019). For Dataset 4, we downloaded the nucleotide alignments of 386 single-copy nuclear genes used in the study by Leebens-Mack et al. (2019), and kept sequences of 115 Chlorophyta, plus four Streptophyta as outgroups. We then used the newly obtained transcriptome assembly from an algal isolate cultured from German clutches of *R. dalmatina* (MVRNA93), and an assembly of North American *Oophila* (reads available from SRA under SRR5445904) from a cultured isolate from the work of Burns et al. (2017). These two transcriptomes corresponded to *Oophila* “Clade A” and “Clade B” of Nema et al. (2019), respectively. We selected 18 genes (Online Resource 1, Table S7) with matches in both transcriptomes, added the respective new transcriptome sequences to the original alignments, and performed codon-based Clustal W alignments in MEGA7 (Kumar et al. 2016). After initial exploratory phylogenetic analyses at the nucleotide level, the alignments were translated to amino acids, yielding a total alignment length of 6758 amino acid (aa) positions for 121 taxa. To improve the analytical power for relationships within or target group (by including additional positions that were difficult to align for more distantly related algae), we ran a second analysis with the same settings for a reduced dataset (Dataset 5) with all taxa from a well-supported clade containing all taxa from Dataset 4 belonging to Chlamydomonadales, Sphaeropleales, Chaetophorales, Oedogoniales, and Chaetopeltidales, as well as representative outgroups (Online Resource 2). The final alignments contained 4219 aa (Dataset 4) and 4892 aa (Dataset 5).

#### Dataset 6

We also assessed the phylogenetic placement of amphibian clutch-associated algae using sequences of the rbcL marker. For this, we first extracted rbcL sequences from the two transcriptomes (MVRNA93 and SRR5445904), then retrieved all rbcL sequences of *Oophila* and *Chlorococcum* from Genbank, plus sequences matching our transcriptome rbcL sequences with 87% (SRR5445904) and 90% (MVRNA93) identity in BLAST searches. To these sequences, we added the metabarcoding rbcL fragments of *Oophila* “Clade B” plus a series of additional algae clearly and in high read numbers associated to *R. dalmatina* clutches.

## Results

**Fig. 2:**
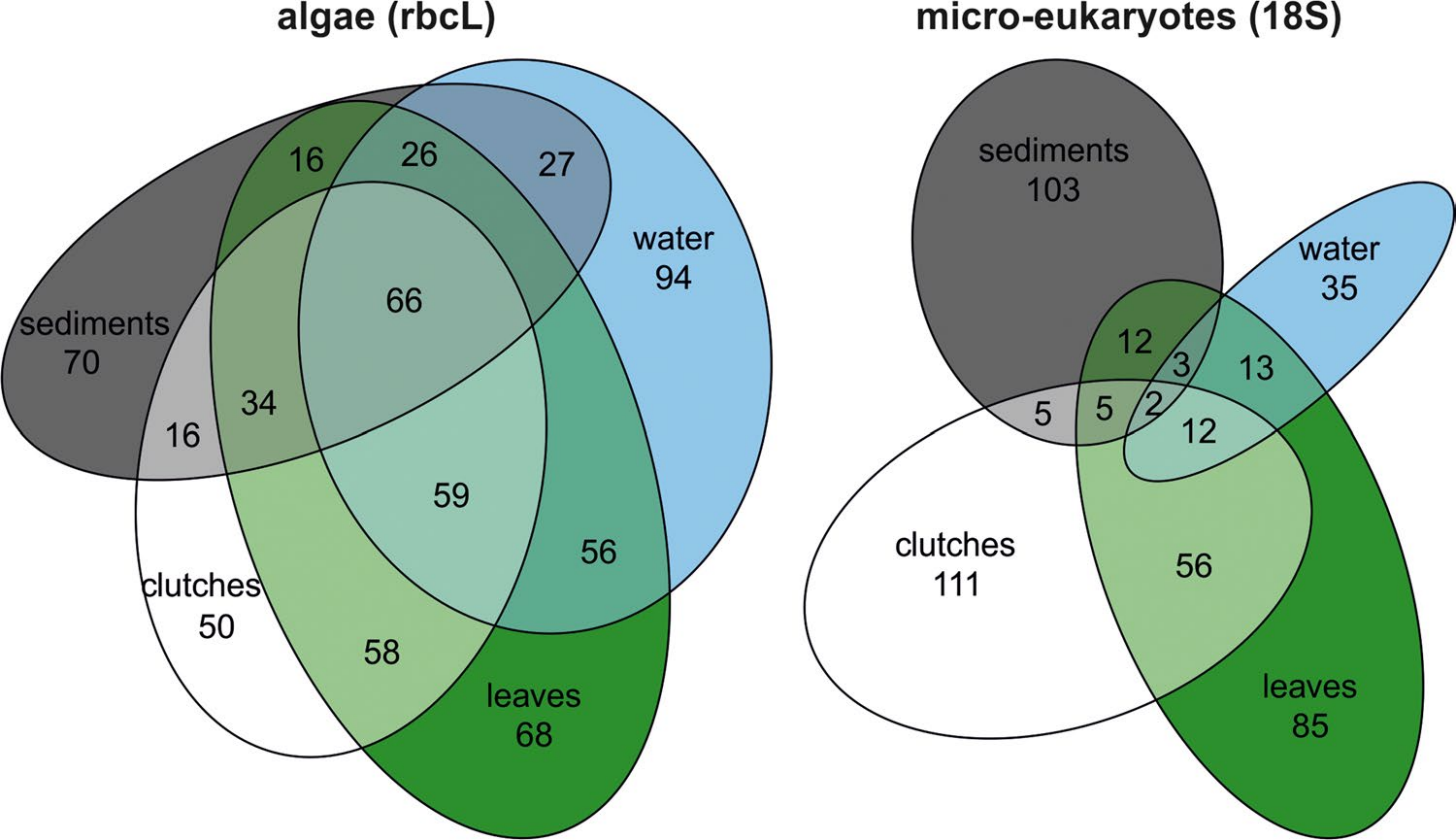
Venn diagram illustrating the distribution of operational taxonomic units (OTUs) across sampling substrates. Fifty rbcL and 111 18S OTUs were found exclusively in *Rana dalmatina* clutch samples. The list of OTUs is included in Online Resource 5

**Fig. 3:**
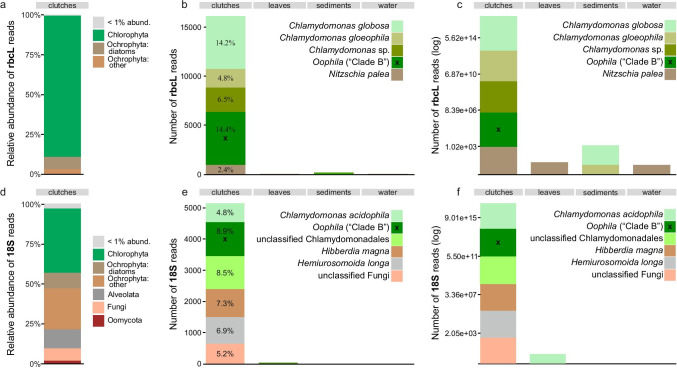
Relative abundance of rbcL (**a**) and 18S (**d**) reads from taxa associated with clutch samples (relative abundance of reads from taxa associated with other substrates in Online Resource 4, Fig. S1). Bar plots for most abundant (sequence abundance) indicator rbcL (**b**, **c**) and 18S (**e**, **f**) operational taxonomic units (OTUs) detected in clutch samples (from Table 1). The y-axis of plots a and c represent sequence counts, while these counts have been log transformed in b and d plots to better highlight the distribution of these OTUs in leaves, sediment, and water samples. For easier interpretation of the graph, the main target taxon (*Oophila*) is marked with x in the respective bars. Percentages in plots b and e represent the relative abundance of sequences for a corresponding taxon in the clutch samples

Overall, DNA metabarcoding resulted in 663 rbcL and 448 18S OTUs (97,724 and 19,130 reads, respectively) across all substrates (water, leaves, sediments, clutches). Water, leaf, and sediment samples contained 351, 383, and 266 rbcL OTUs, and 71, 188, and 133 18S OTUs, respectively. Clutch samples harbored 306 rbcL and 195 18S OTUs. The proportion of these OTUs found exclusively in clutch samples was 16.3% (50 OTUs) and 56.9% (111 OTUs) for rbcL and 18S data, respectively (Fig. 2; Online Resource 1, Table S3). In addition to green algae assigned to Chlorophyta (89% of reads), rbcL OTUs also included taxa from diatoms (Bacillariophyceae; 7.8%) and other Ochrophyta (3.1%) in the clutch samples (Fig. 3a). The relative abundance of 18S reads associated with clutch samples was 40.6% for Chlorophyta, 26% for non-diatom Ochrophyta, and 9.5% for diatoms. Besides the latter, clutch samples contained taxa from Alveolata (12.1% of reads), Fungi (7.7%), and Oomycota (1.9%) in the 18S data set (Fig. 3d). Most frequently detected and most abundant (sequence read abundance) OTUs in the clutch samples were taxa from *Chlamydomonas*, *Nitzschia,* and *Oophila* as suggested by the rbcL metabarcoding data (Fig. 3b, c; Online Resource 1, Table S3), but also taxa from Alveolata and non-diatom Ochrophyta as indicated by the 18S data (Fig. 3e, f; Online Resource 1, Table S3) that were infrequent in other environmental samples (Table 1).

**Table 1:** The ten most frequently occurring and abundant (sequence abundance) rbcL and 18S OTUs in *Rana dalmatina* clutch samples. Note that the marked taxa in the table represent blastn first match where the percentage in between parentheses denote the blastn identity percentage to noted taxa. Asterisks (*) indicate that the OTU was identified as an indicator OTU for clutch samples. An OTU with double asterisk (**) was identified as an indicator OTU for clutch + leaf samples (see Online Resource 3 for all indicator OTUs). Frequency denotes the occurrence number of the OTU across clutches samples (total = 28 samples). Rel. abund. denotes relative abundance of sequences of the given OTU in clutches samples. Specificity and sensitivity (range 0–1) denote the probability that the OTU is associated with the clutches samples, and indicate the probability of finding the OTU in clutches samples, respectively

	Frequency	Rel. abund	Specificity	Sensitivity
rbcL OTUs				
Chlamydomonadaceae; *Chlamydomonas gloeophila* (99.4%)*	19	14.2%	0.994	0.679
Chlamydomonadaceae; *Chlamydomonas globosa* (92.2%)*	19	4.8%	0.971	0.679
Bacillariophyceae; *Nitzschia palea* (100%)*	19	2.4%	0.965	0.679
Chlamydomonadaceae; *Oophila* sp. (99.7%)*	18	14.4%	1	0.643
Pleurastraceae; *Pleurastrum* sp. (94.3%)*	18	1.1%	0.844	0.643
Chlamydomonadaceae; *Chlamydomonas* sp. (93.8%)**	17	2.6%	0.531	0.607
Chlamydomonadaceae; *Chlamydomonas* sp. (97.3%)*	14	6.5%	1	0.500
Chlorellaceae; *Nannochloris* sp. (89.9%)	14	2.2%	0.350	0.500
Trebouxiophyceae; *Choricystis* sp. (97.3%)	13	17.1%	0.283	0.464
Ulotrichaceae; *Gloeotilopsis planctonica* (86%)	10	3.1%	0.278	0.357
18S OTUs				
Unclassified Chlamydomonadales (98.4%)*	15	8.5%	1	0.536
Unclassified Bacillariophyceae (99.2%)*	15	2.0%	0.950	0.536
Alveolata; *Hemiurosomoida longa* (99.2%)*	14	6.9%	1	0.500
Ochrophyta; *Mallomonas* sp. (99.2%)*	13	4.2%	0.888	0.464
Chlamydomonadaceae; *Chloromonas oviformis* (100%)*	13	2.3%	0.827	0.464
Chlamydomonadaceae; *Chloromonas subdivisa* (100%)*	13	2.1%	0.881	0.464
Chlamydomonadaceae; *Chlamydomonas acidophila* (100%)*	10	4.8%	0.980	0.357
Chlamydomonadaceae; *Oophila* sp. (100%)*	6	8.9%	1	0.214
Ochrophyta; *Hibberdia magna* (92.1%)*	6	7.3%	1	0.214
Unclassified Fungi (93.9%)*	5	5.2%	1	0.179

**Fig. 4:**
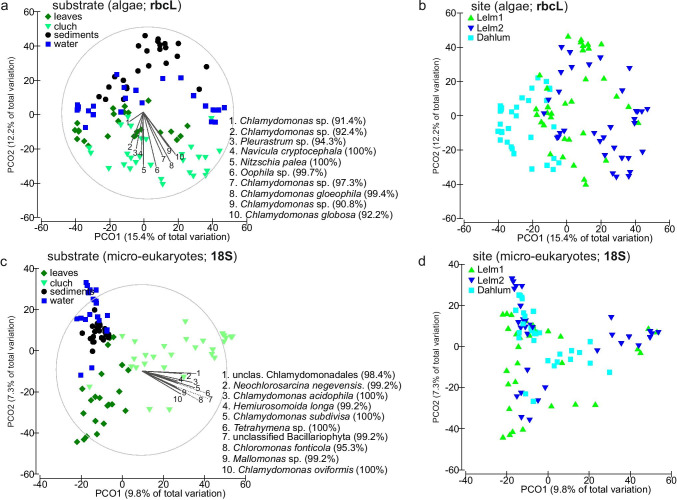
Principal coordinates analysis (PCO) ordination plots (based on Bray–Curtis matrices) for algal (rbcL; **a**–**b**) and micro-eukaryotic (18S; **c**–**d**) communities from all sampled substrates and sites. Vectors in a and c plots denote (Spearman) correlation vectors of 10 most important indicator operational taxonomic units (OTUs) in *Rana dalmatina* clutch samples. The percentage in the parentheses after indicator OTU represent the blastn identity percentage for that taxon (i.e. first blastn match)

PERMANOVA analyses revealed significant effects of substrate, sampling site, and sampling date for both algal (rbcL) and micro-eukaryotic (18S) communities (Table 2; Fig. 4). The highest proportion of variance was explained by the substrate (Table 2), followed by the sampling site for algal (rbcL) communities, but by sampling date for micro-eukaryotic (18S) communities (Table 2).

**Table 2:** PERMANOVA (Type I SS, reads per sample as a covariate) results for rbcL and 18S metabarcoding data sets (based on operational taxonomic units (OTU) tables, Online Resource 5) to test the effect of ‘substrate’, ‘sampling site’ and ‘sampling date’ on the algal (rbcL) and micro-eukaryotic (18S) communities. Tests were performed using the Bray–Curtis similarity matrix based on the Log-transformed sequence data, as well as on the UniFrac distance matrix based on the Maximum-likelihood phylogenetic trees of OTUs. Factor ‘substrate’ includes four categories: samples of leaves (*n* = 24), sediments (*n* = 24), water (*n* = 24) and clutches (*n* = 28). Factor ‘sampling site’ includes three categories: Lelm1 (*n* = 34), Lelm2 (*n* = 34), Dahlum (*n* = 32). Factor ‘sampling date’ includes nine categories: 25-Feb-19 (*n* = 9), 12-Mar-19 (*n* = 9), 25-Mar-19 (*n* = 15), 28-Mar-19 (*n* = 15), 05-Apr-19 (*n* = 15), 11-Apr-19 (*n* = 15), 18-Apr-19 (*n* = 13), 13-Jun-19 (*n* = 9). Factor values after the covariate indicate test results when the respective factor was included in the model as the last one (Type I analyses)

		Bray–Curtis matrix			UniFrac matrix		
	df	R^2^	pseudo-F	*P*-value	R^2^	pseudo-F	*P*-value
Factor (rbcL)							
Covariate(reads)	1	0.032	7.325	< 0.001	0.024	5.1404	0.006
Substrate	3	0.205	15.924	< 0.001	0.176	12.472	< 0.001
Sampling site	2	0.153	17.751	< 0.001	0.112	11.903	< 0.001
Sampling date	7	0.076	2.511	< 0.001	0.066	2.0197	0.005
Factor (18S)							
Covariate(reads)	1	0.037	10.209	< 0.001	0.047	11.499	< 0.001
Substrate	3	0.146	13.383	< 0.001	0.135	11.028	< 0.001
Sampling site	2	0.072	9.867	< 0.001	0.050	6.0527	< 0.001
Sampling date	7	0.075	2.944	< 0.001	0.074	2.5864	< 0.001

**Fig. 5:**
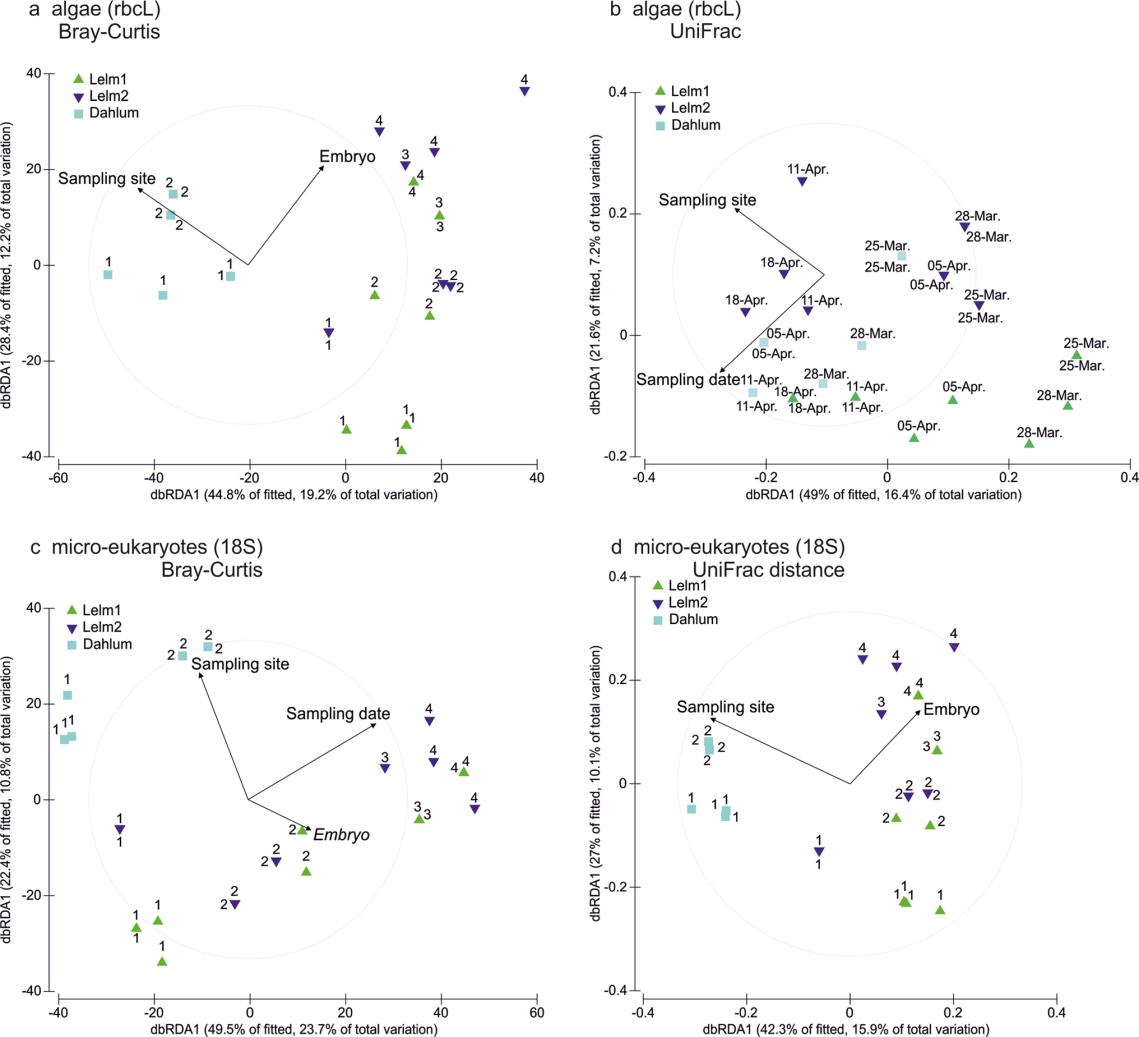
Distance-based redundancy analysis (dbRDA) ordination plots for algal (rbcL; **a**–**b**) and micro-eukaryotic (18S; **c**–**d**) communities in *Rana dalmatina* clutch samples as based on Bray–Curtis similarity (a, c) and UniFrac (b, d) distance matrices. Vectors on the plots represent the most important variables for the community compositions of algae (rbcL) and micro-eukaryotic (18S) associated with the clutches as based on DistLM analyses. Numbers above individual dots denote the estimated embryo developmental stage categories. Dates above individual dots denote sampling dates. Two values associated with one dot indicate the two overlapping dots with the same coordinates

Based on DistLM analysis, the best predictors for algal (rbcL) communities associated with the clutch samples were sampling site and embryo developmental stage category using the Bray–Curtis similarity matrix, but sampling site and sampling date for the UniFrac distance matrix (Online Resource 1, Table S4, Fig. 5). Sampling date, however, correlates significantly with embryo developmental stage (Spearman R > 0.950, *P* < 0.015 for all ponds) and water temperature (Spearman *R* > 0.890, *P* < 0.007; Online Resource 4, Fig. S2). Best predictors for micro-eukaryotic (18S) communities associated with the clutch samples were also sampling site and embryo developmental stage, but additionally, sampling date was an important predictor in the analyses with Bray–Curtis similarity matrix (Online Resource 1, Table S4; Fig. 5). Pairwise analyses between sampling site and substrate showed that clutch samples differed between all sites for both algal and micro-eukaryotic communities (*P* < 0.033 for all cases, except for 18S UniFrac data set between sites of Lelm1 and Lelm2; Online Resource 1, Table S5). Also, algal and micro-eukaryotic communities from other substrates (water, leaves, sediments) varied significantly among sampling sites (Online Resource 1, Table S5).

Indicator species analyses (samples not separated by site) identified 22 algal (rbcL) and 30 micro-eukaryotic (18S) OTUs that were characteristic for *R*. *dalmatina* clutch samples (Online Resource 3; interactive Krona chatrs in Online Resource 4, Figs. S3, S4). OTUs assigned to Chlamydomonadaceae (*Chlamydomonas* sp., *Oophila* sp., *Chloromonas* sp.) accounted for 64% (14 OTUs) and 20% (6 OTUs) of indicator taxa in the rbcL and 18S data sets, respectively. Other indicator taxa for clutch samples included OTUs assigned to other Chlamydomonadales (18%, 4 OTUs) and Bacillariophyceae (diatoms; 18%, 4 OTUs) in the rbcL data set (Online Resource 4, Fig. S3); and other Chlamydomonadales (10%, 3 OTUs), Chlorosarcinaceae (3%, 1 OTU), Bacillariophyceae (13%, 4 OTUs), other Stramenopiles (30%, 9 OTUs), Fungi (10%, 3 OTUs), Chiliophora (10%, 3 OTUs) and Euglenozoa (3%, 1 OTU) in the 18S data set (Online Resource 4, Fig. S4).

Since PERMANOVA showed significant differences in OTU composition between sampling sites (Table 2, Fig. 4), indicator species analyses were also performed separately for the *R. dalmatina* clutch samples from each site (Lelm1, Lelm2, Dahlum). The analyses revealed 21, 12, and 6 algal (rbcL) OTUs from the three sampling sites, respectively, that were identified as characteristic to clutch samples (Online Resource 3). For the same sites, indicator species analyses for micro-eukaryotes (18S data) found 6, 18, and 25 indicator OTUs from clutch samples (Online Resource 3). The PERMANOVA analyses, only with indicator OTUs for clutch samples, also demonstrated the significant effect of sampling site (R^2^ = 0.357, *P* < 0.001 and R^2^ = 0.299, *P* < 0.001 for algae and micro-eukaryotes, respectively; Online Resource 4, Fig. S5). In general, the ponds that were close together (i.e., the two proximate sampling sites of Lelm1 and Lelm2) shared a higher number of indicator OTUs from clutch samples (Online Resource 4, Fig. S6), thus had more similar communities (Online Resource 4, Fig. S5).

**Fig. 6:**
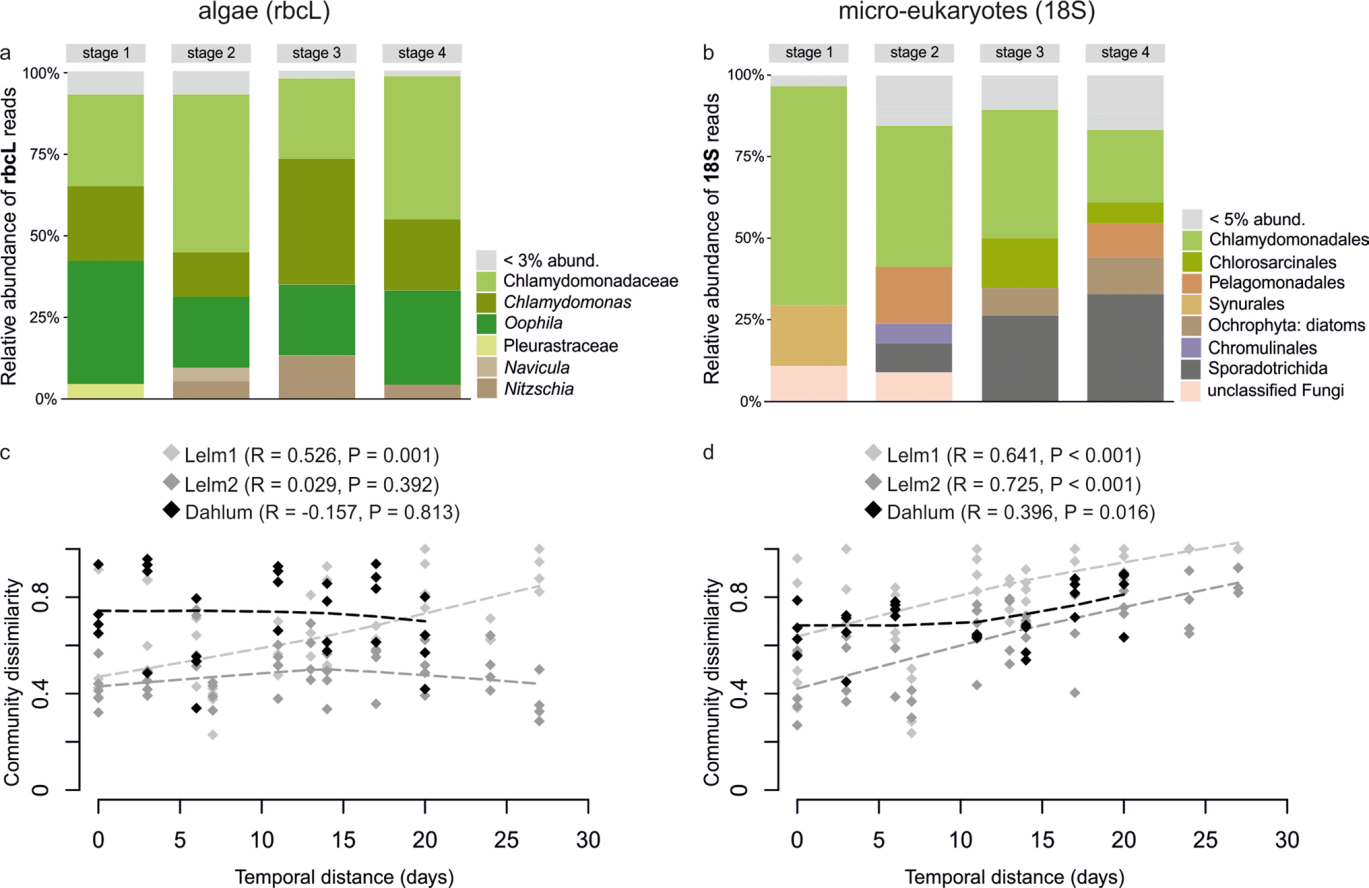
Relative abundance of reads for indicator OTUs in the pooled *Rana dalmatina* clutch samples across embryo developmental stage categories (**a**–**b**), and relationships between *R. dalmatina* clutch-associated operational taxonomic units (OTUs) community dissimilarity and temporal distance for each sampling site (**c**–**d**). OTUs annotated to order level for 18S data (a) and to genus level for rbcL indicator OTUs (b). Higher level taxa in b plot indicate that blastn match was lower than 95%, thus the OTU was not annotated to genus level

Taxa associated with *R. dalmatina* clutches also displayed temporal distance decay of similarity patterns (Fig. 6). This was statistically significant for the algal (rbcL) communities in the sampling site Lelm1, whereas temporal distance did not affect the similarity between clutch samples from other sites (Lelm2 and Dahlum; Fig. 6c). Micro-eukaryotic (18S) communities associated with clutch samples were subjected to temporal distance decay of similarity at all sampling sites (Fig. 6d). The temporal distance decay of similarity patterns for algal and micro-eukaryotic communities in the clutch samples displayed the same patterns when including only indicator OTUs to the temporal distance decay analyses (Online Resource 4, Fig. S7). Temporal distance decay patterns were also demonstrated by the communities associated with water and leaf samples but not with sediment samples (Online Resource 4, Fig. S8).

**Fig. 7:**
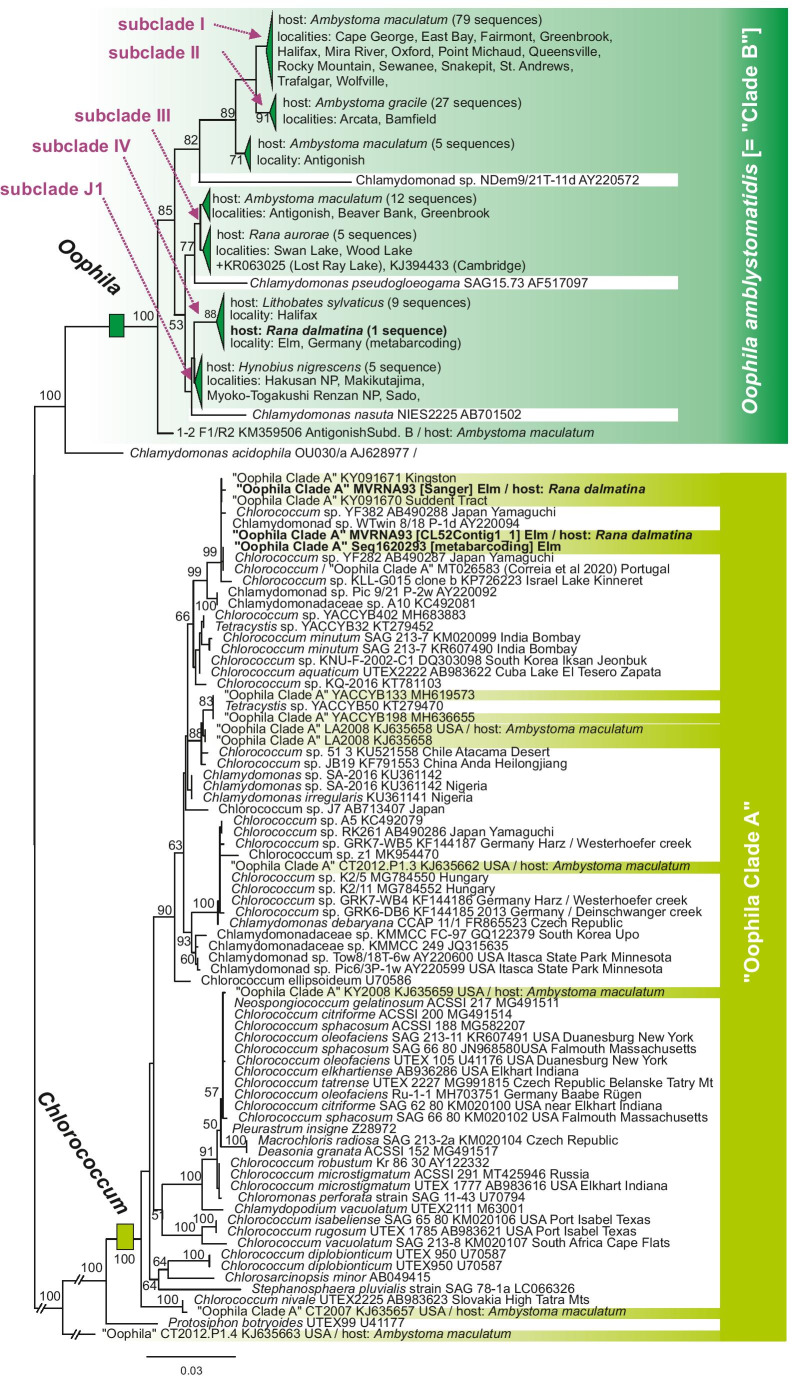
Maximum Likelihood tree inferred from DNA sequences of the 18S rRNA gene (Dataset 3; 1878 bp) comprising all sequences assigned to the *Oophila* clade and the *Chlorococcum* clade in the exploratory analysis of Dataset 1 (Online Resource 4, Fig. S9). Terminals with identical or very similar sequences have been merged; see Online Resource 4, Fig. S11 for an expanded tree with all terminals and their Genbank accession numbers. Dark green marks the clade of amphibian-associated *Oophila* (“Clade B” according to Nema et al. 2019) with subclades according to Kim et al. (2014) and Muto et al. (2017), light green marks the *Oophila*-like lineages found associated to amphibians within the *Chlorococcum* clade (“Clade A” according to Nema et al. 2019). Numbers at nodes show bootstrap values in percent (only shown if > 50%, and removed from the shallowest nodes for better graphical representation)

**Fig. 8:**
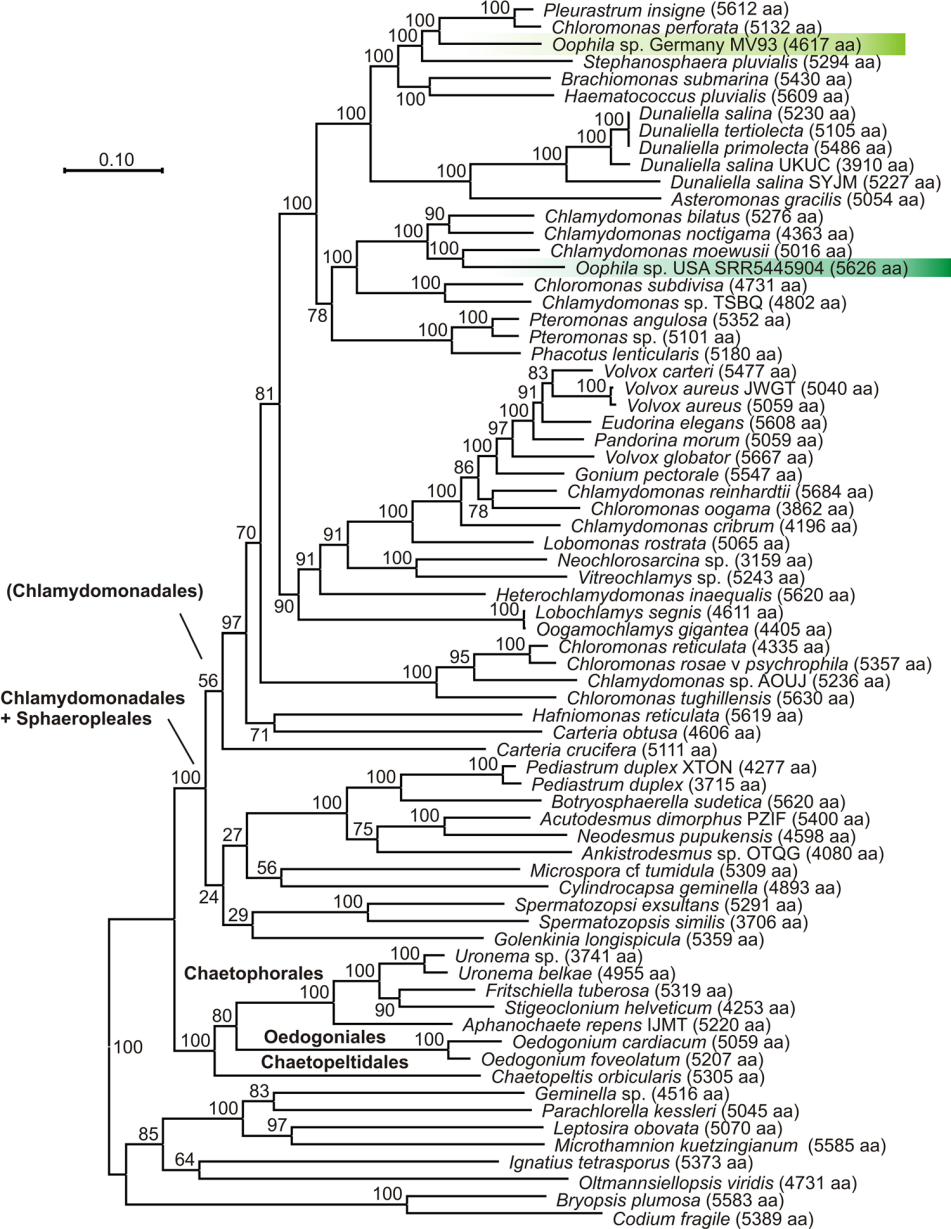
Maximum Likelihood tree inferred from DNA sequences of 18 nuclear protein-coding genes after exclusion of hypervariable regions (Dataset 5; alignment length 4892 amino acids), comprising all Chlamydomonadales and related taxa for which sequences were available from the study of (Leebens-Mack et al. 2019), plus sequences extracted from transcriptomes of one *Oophila* strain isolated from a clutch of *Ambystoma maculatum* (dark green; corresponding to “Clade B” of Nema et al. (2019)) and of one *Oophila*-like representative of the *Chlorococcum* clade isolated from a clutch of *Rana dalmatina* (light green; corresponding to “Clade A” of Nema et al. (2019)). Numbers at nodes show bootstrap values in percent. See Online Resource 4, Fig. S13 for an extended tree containing all available taxa within Chlorophyta

An exploratory analysis of all sequences of the 18S rRNA gene from previous studies targeting *Oophila* (Correia et al. 2020; Kim et al. 2014; Muto et al. 2017; Nema et al. 2019) confirmed that strains assigned to *Oophila* in these studies belong to two distinct clades within Chlorophyta (Dataset 1; Online Resource 4, Fig. S9). A closer look specifically at these two clades (Dataset 3; Fig. 7) reconstructs those sequences associated to amphibian clutches considered as “Clade B” by Nema et al. (2019) as a monophyletic group. This group contains only a few isolates of free-living algae besides the clutch-associated ones, whereas the sequences of “Clade A” sensu Nema et al. (2019) are placed at different positions within a clade containing numerous free-living species of *Chlorococcum*. Most of the clutch-associated isolates in this clade have 18S sequences identical or extremely similar to free-living strains of different *Chlorococcum* species, and the tree identifies seven independent, not directly related *Chlorococcum*-like lineages isolated from amphibian clutches. Samples associated to *R. dalmatina* clutches in Germany are found in both main clades: the 18S sequences identified as *Oophila* in our metabarcoding study cluster within “Clade B”, whereas one isolate cultured from a clutch in 2018 is placed in “Clade A” (Fig. 7). The latter is nearly identical (99.9%) in 18S sequence to Canadian isolates from Sudden Tract (spelled Suddent Tract in the respective Genbank records; KY091670) and Kingston, Ontario (KY091671). Phylogenetic analysis based on a comprehensive multigene data set (Fig. 8) confirms with maximum support that representatives of the two main clades are not closely related to each other and belong to phylogenetically distant groups of green algae.

An analysis of the metabarcoding OTU representative 18S sequences along with selected sequences of green algae (Dataset 2; Online Resource 4, Fig. S10) revealed that in our study ponds in the Elm region, clutches of *R. dalmatina* were colonized by a plethora of green algae of distinct phylogenetic positions. Forty-eight OTUs (18S data) of green algae identified from clutches were included in the tree; besides *Chlorococcum* and *Oophila*, some of these matched reference sequences of algae as diverse as *Chlamydomonas, Chloromonas, Chlorosarcina, Planophila,* and *Tetracystis*. Moreover, the rbcL data contained 214 clutch-associated green algal OTUs. Besides *Chlamydomonas* and *Oophila* (Fig. 2a, b), other frequently occurring green algae were assigned to genera such as *Microthamnion*, *Nannochloris,* and *Choricystis*. Since the rbcL primers used herein target only a few algal groups, but 18S primers are universal across a wide range of micro-eukaryotes, the detected clutch-associated green algal communities varied between these two markers. Clutch-associated green algal genera detected with rbcL and 18S are outlined in Online Resource 4, Fig. S12. The rbcL phylogenetic analysis (Dataset 6; Online Resource 4, Fig. S14) did not resolve any of the deeper nodes in the green algae tree with bootstrap support but further confirmed that “Clade A” is a paraphyletic assemblage of isolates related to different free-living green algae.

## Discussion

### Amphibian clutches as ecosystem for algae and other micro-eukaryotes

In our study, DNA metabarcoding revealed that *R. dalmatina* clutches in the Elm region (Germany) provide a distinct ecosystem for an array of algae and other micro-eukaryotes. More than 300 algal OTUs (rbcL marker) and nearly 200 micro-eukaryotic OTUs (18S marker) were detected in the clutch samples. In contrast to the recent metabarcoding study of the North American salamander *Ambystoma maculatum* egg capsules*,* where Jurga et al. (2020) found no evidence for algal lineages outside the ‘*Oophila*’ clade (according to Kim et al. 2014) colonizing the salamander’s eggs, our data contained a wide range of algae associated to *R. dalmatina* clutches. Because the egg capsules of *R*. *dalmatina* are less compact compared to those of *A. maculatum*, especially in later stages of development, it is difficult to isolate exactly the inner capsule. Therefore, the clutch samples in our study may be prone to an inevitable ‘environmental contamination’ with algae that are not strictly inside the egg capsule. Some of the detected taxa may therefore occur on the surface of the clutch or in cavities between egg capsules rather than within the capsular chambers directly surrounding the eggs.

Besides green algae, we often detected diatoms in clutch samples of *R. dalmatina*. Marine diatoms are known to live in the gelatinous egg masses of polychaetes (Chaffee and Strathmann 1984; Strathmann 2000) and mollusks (Biermann et al. 1992; Cohen and Strathmann 1996). This symbiotic relationship is likely beneficial for both partners (Peyton et al. 2004), but so far, no studies on the colonization of diatoms of clutches of *Rana* or other amphibians have been published. The benthic diatoms *Nitzschia palea*, *Navicula cryptocephala,* and *N. tripunctata* detected in our study are widespread and common species (Lange-Bertalot et al. 2017). It is very likely that they use *R. dalmatina* clutches as a substrate (micro-habitat), i.e., they live on them and not inside, which may speak against a close symbiotic relationship.

Nevertheless, the identified algal and other micro-eukaryotic communities associated with frog clutches had very distinctive compositions compared with the communities in other environmental samples in our study (Fig. 4), with more than 20 indicator OTUs in this micro-habitat (Online Resource 3). Although we sampled seemingly similar ponds, we found a relatively strong effect of the location on algal and micro-eukaryotic community structures, similar to observations by Jurga et al. (2020). Moreover, in an exploratory metabarcoding study of clutches of the related host *Rana parvipalmata* from a pond in northwestern Spain, with very clear water on granitic soil, the rbcL and 18S data of samples processed in exactly the same way revealed no traces of any other green algae except *Oophila,* and very few additional micro-eukaryotes (data to be in-depth analyzed in future studies; list of OTUs in Online Resource 1, Table S6). This suggests that the composition of the community colonizing amphibian clutches can drastically differ depending on environmental properties. The ponds in the Elm are rather nutrient-rich, on substrate characterized by limestone components and with often turbid water and muddy bottom. This is possibly favoring the growth of numerous green algae and other micro-eukaryotes, whereas nutrient-poor environments (like that of the *R. parvipalmata* clutches sampled with identical methodology; Online Resource 1, Table S6) may harbor a less diverse reservoir of potential clutch colonizers. Moreover, we observed a temporal distance decay of similarity of the clutch-associated communities, which most probably was linked to changes in the environment over time (Online Resource 4, Fig. S8). This further confirms that local conditions are affecting the clutch-associated communities of algae and other micro-eukaryotes. As stated before, probably not all clutch-associated taxa identified in our study colonize the inner egg capsule. Yet, it appears that, depending on the habitat characteristics, amphibian clutches may attract numerous species of algae and other micro-eukaryotes outside of the ‘*Oophila*’ clade(s) to exploit this micro-habitat.

### rbcL vs. 18S gene markers for metabarcoding

Overall, we found comparable community structuring patterns between rbcL *vs*. 18S metabarcoding data (Fig. 5, Table 2). However, as expected, the taxonomic resolution varied between markers (Figs. 2 and 3, Table 1). The universal 18S primers target a broad range of micro-eukaryotic linages, whereas rbcL primers capture a subset of photosynthetic micro-algae, which presumably only represent a subset of the micro-eukaryotes detected with the 18S marker. Furthermore, by applying the same clustering threshold (97%), the number of OTUs was higher in the rbcL metabarcoding data set. In the clutch samples, more than 200 rbcL OTUs were identified as green algae, but less than 50 green algal OTUs were identified via 18S amplicons. It is acknowledged that the choice of primers affects the results of biodiversity assessments (Hajibabaei et al. 2019; Horton et al. 2017; Piñol et al. 2019; Tedersoo et al. 2015). The relatively conserved 18S gene allows designing rather universal primers across multiple taxonomic groups; however, with the universality, there is an expected loss in resolution. The low variability within the 18S gene has been demonstrated for multiple taxonomic groups (Anslan and Tedersoo 2015; Tang et al. 2012), including green algae (Hall et al. 2010). Therefore, the lower richness of green algae associated with the frog clutches in the 18S data set could partly result from the low resolution of that marker gene compared to rbcL, which possesses higher variability among green algae (Hall et al. 2010). Nevertheless, the 18S data set contained some green algal genera (alongside other micro-eukaryotes) that were not identified via rbcL amplicons (Online Resource 4, Fig. S12). Wider variety of detected taxa demonstrates the usefulness of multi-marker approaches, which has also been recognized in several other metabarcoding studies (Adamowicz et al. 2019; da Silva et al. 2019; Zhang et al. 2018).

### Occurrence and relationships of amphibian clutch-associated green algae in Europe

Isolates of *Oophila amblystomatis* associated to amphibian clutches have so far been identified by molecular means from North America and Japan (Kim et al. 2014; Muto et al. 2017; Nema et al. 2019). Our study provides the first molecular confirmation of the occurrence of *Oophila* from Central Europe where the occurrence of green algae in clutches of *Rana dalmatina* and *R. temporaria* has previously been reported based on microscopic examination (Baumgartner et al. 1996; Fernández de Larrea González 2018). Interestingly, Baumgartner et al. (1996) observed that *R. dalmatina* clutches were more intensively colonized by green algae than *R. temporaria* clutches, even under sympatric occurrence of the two host species.

One of the most common green alga (Table 1) in our 18S metabarcoding data set from *R. dalmatina* clutches matched sequences commonly assigned to *Oophila* (e.g., Jurga et al. 2020; Kerney 2011; Kerney et al. 2011; Kim et al. 2014; Muto et al. 2017) and considered as “Clade B” by Nema et al. (2019) (Fig. 7). Algae belonging to *Oophila* “Clade B” have rarely been found free-living: as one of the few examples, Lin and Bishop (2015) detected them via environmental DNA sequencing from amphibian breeding ponds in North America, and the respective 18S clade in Fig. 7 contains sequences from a few further isolates available in Genbank and flagged as free-living (named as unidentified chlamydomonad, *Chlamydomonas nasuta*, and *C. pseudogloegama*, respectively). In our study, OTUs assigned to *Oophila* “Clade B” (with > 97% sequence similarity) were not detected from other environments, confirming that this clade mostly contains strict symbionts associated to amphibian clutches that are infrequent outside of this habitat.

Within this clade, previous studies (Kim et al. 2014; Muto et al. 2017) defined several subclades with a certain degree of host-specificity: subclade I was isolated from the salamander *Ambystoma maculatum*, subclade II from *A. gracile*, subclade III from the frog *Rana aurora* and from *A. maculatum*, subclade IV from the frog *Lithobates sylvatica*, and subclade J1 from the salamander *Hynobius nigrescens*. The “Clade B” 18S sequence from our metabarcoding data set clustered with subclade IV without sequence differences to North American sequences (Fig. 7). Although based on very short DNA sequences only, this provides preliminary evidence for a widespread occurrence of *Oophila* strains across the Holarctic.

Our metabarcoding data revealed numerous other algae in *R. dalmatina* clutches, many of which we consider as opportunistic colonizers of these egg masses. Several of these, however, have sequences matching those of algae considered to belong to *Oophila* “Clade A” according to Nema et al. (2019) (Online Resource 4, Fig. S10). Full 18S sequences from a culture isolated from an *R. dalmatina* clutch (isolate MVRNA93), both assembled from a transcriptome and obtained via Sanger sequencing, fully matched sequences KY091670-KY091671 from two Canadian “Clade A” samples (Fig. 7). This suggests that also green algae strains belonging to “Clade A” are widely distributed across both the Nearctic and Palearctic and readily colonize amphibian egg clutches.

However, given the relative scarcity of “Clade A” algae in metabarcoding studies from clutches, including our results herein, we assume that most of the reported benefits from the mutualistic algae-amphibian relationship refer to “Clade B” algae (e.g., Gilbert 1942; Gilbert 1944; Pinder and Friet 1994; Small and Bishop 2020 — all of whom examined *A. maculatum*). Whether a mutualistic relationship is maintained by “Clade A” algae remains to be seen. Anderson et al. (1971) and Bachmann et al. (1986) both found *A. tigrinum* embryonic mortality was positively correlated with egg capsule algae (but see Hutchison 1971 for a rebuttal). It is possible that these differences in symbiotic relationship are not due to the host salamander (*A. tigrinum vs*. the more commonly studied *A. maculatum*), but to the relative portion of “Clade A” *vs*. “Clade B” algae. Controlled co-culture studies with the different clades would help resolve these potentially dynamic interspecific relationships.

### Identity of Oophila amblystomatis

Combining our DNA metabarcoding and phylogenetic results allows us to draw conclusions on the identity of *O. amblystomatis* and, thereby, the monotypic genus *Oophila* itself. Most recent studies have considered isolates phylogenetically belonging to “Clade B” to represent this genus (e.g. Kerney 2011; Kerney et al. 2011; Kim et al. 2014; Muto et al. 2017). However, Nema et al. (2019) suggested that “Clade A” may represent this genus, based on analysis of numerous isolates, some of which were obtained from amphibian clutches collected close to the presumptive type locality of *O. amblystomatis*, Middlesex Fells in Massachusetts. *O. amblystomatis* (Lambert ex Wille 1909) species was originally named informally by F.D. Lambert in 1905 based on samples of algal cells collected and preserved from *Ambystoma maculatum* embryos (at the time called *Amblystoma punctatum*), but no genetic information or living type strain exists from the original materials. While a final resolution of the conundrum can only be achieved after careful examination of all historical evidence, including Lambert's original materials (curated by Craig Schneider at Trinity College, Connecticut, according to information in Nema et al. 2019, who erroneously wrote about type species rather than type specimen when referring to this material), the results of the present study suggest that algae more strictly and more frequently associated to amphibian clutches belong to “Clade B”. Therefore, we hypothesize that Lambert's observations also referred to representatives of this clade.

As a first line of evidence, our initial efforts to isolate algae from *R. dalmatina* clutches in the Elm yielded one isolate (MVRNA93) belonging to “Clade A” and identical in its 18S sequence to two Canadian samples (KY091670, KY091671) from the work of Nema et al. (2019). No isolate belonging to “Clade B” was obtained. However, according to the DNA metabarcoding data, the “Clade A” alga is exceedingly rare in the *R. dalmatina* clutches, whereas “Clade B” algae (here considered as *Oophila*) had the highest read numbers of any algal taxon in the clutch samples. Therefore, it is likely that “Clade B” algae are more difficult to culture than (many) “Clade A” algae, and the latter are, therefore, more successfully isolated and cultured (or have a high likelihood to contaminate other isolates, even if present in only very minor proportion in a sample). Two other recent studies are in agreement with this hypothesis: firstly, the DNA metabarcoding study of Jurga et al. (2020) only found “Clade B” sequences in numerous North American salamander clutches studied; and secondly, Correia et al. (2020) obtained a free-living “Clade A” isolate from a pond in Portugal, which they specifically characterize as robust and with promising growth performance even at industrial scale.

Secondly, our phylogenetic analysis (Fig. 7), including all 18S sequences assigned to *Oophila* in previous studies plus sequences of other algae with BLAST matches, suggests that “Clade A” is a conglomerate of not directly related algae, numerous samples of which are identical or near-identical with isolates of free-living algae of the genus *Chlorococcum*. This is particularly obvious for two isolates from *Ambystoma maculatum* clutches: sequence KJ635657 is identical to a sequence of *C. nivale* (AB983623), and KJ635659 agrees with sequences of numerous nominal *Chlorococcum* species such as *C. citriforme, S. sphacosum, C. oleofaciens, C. elkhartiense,* and *C. tatraense*. For other amphibian clutch-associated sequences of “Clade A”, matching sequences are from unnamed free-living *Chlorococcum* originating from a diverse array of countries and continents (Chile, China, Czech Republic, Germany, Hungary, Israel, Japan, Portugal, Slovakia), suggesting these are ecological generalists occurring globally.

Upon finding sequences of *Oophila* “Clade A” clustering with *Chlorococcum*, Correia et al. (2020) suggested transferring *O. amblystomatis* to the genus *Chlorococcum*, and treated the species as *Chlorococcum amblystomatis*. While we agree that “Clade A” algae should be assigned to *Chlorococcum*, we disagree with the conclusion regarding the species name *amblystomatis*. Instead, we suggest that it is biologically more probable and taxonomically more parsimonious to assign the nomen *O. amblystomatis* to the algae in “Clade B”, which based on DNA metabarcoding have been found to be numerically most abundant in amphibian clutches in North America (Jurga et al. 2020), Europe (this study) and potentially Japan (Muto et al., 2017). “Clade B” appears to be more strictly associated to amphibian clutches than those algae of “Clade A”, which may only opportunistically and occasionally colonize this micro-environment. According to our phylotranscriptomic analysis, “Clade B” appears to belong to a clade mostly consisting of *Chlamydomonas* rather than *Chlorococcum* species, and a final conclusion on the genus name *Oophila* will therefore only be possible after a more comprehensive taxonomic revision of the Chlamydomonadales.

## Supplementary Information

Below is the link to the electronic supplementary material.

Supplementary file1 (XLSX 62 KB)

Supplementary file2 (PDF 153 KB)

Supplementary file3 (XLSX 104 KB)

Supplementary file4 (PDF 4184 KB)

Supplementary file5 (XLSX 692 KB)

Supplementary file6 (R 3 KB)

## Data Availability

Raw Illumina MiSeq metabarcoding data is deposited in the Sequence Read Archive (SRA), BioProject ID: PRJNA714784. Raw Illumina NextSeq metatranscriptomics data is deposited in SRA, BioProject ID: PRJNA712983. The 18S sequence of an algal isolate MVRNA93 from *Rana dalmatina* clutch has been deposited in Genbank, under accession number MW723501.

## Code availability

Herein used R codes are available in Online Resource 6.
