# Supplementary material for: Diversity and substrate-specificity of green algae and other micro-eukaryotes colonizing amphibian clutches in Germany, revealed by DNA metabarcoding
Source: Naturwissenschaften. 2021 Jun 28;108(4):29. doi: 10.1007/s00114-021-01734-0 (PMC8238718; doi:10.1007/s00114-021-01734-0)
Supplement: Supplementary file 2 — Supplementary file2 (PDF 153 KB) [file 114_2021_1734_MOESM2_ESM.pdf]

Diversity and substrate-specificity of green algae and other micro-eukaryotes colonizing amphibian clutches in Germany, revealed by DNA metabarcoding

Sten Anslan[#], Maria Sachs, Lois Rancilhac, Henner Brinkmann, Jörn Petersen, Sven Künzel, Anja Schwarz, Hartmut Arndt, Ryan Kerney, Miguel Vences

#Corresponding author: s.anslan@tu-braunschweig.de; Zoological Institute, Technische Universität Braunschweig, Braunschweig, Germany

Journal: The Science of Nature

Supplementary methods (extended methods)

Text in color denote the parts that are not in the main text of the manuscript

Sampling

Sampling was performed at three small ponds in Germany, all located in the Elm region near Braunschweig, here named Lelm1, Lelm2, and Dahlum (Online Resource 1, Table S1; Fig 1a). Samples were collected from four types of substrates: 1) water, 2) sediment, 3) tree leaves from the bottom of the pond, and 4) *Rana dalmatina* clutches (Fig. 1b). Water samples were collected via an algal net (0.25 µm mesh) by filtering and concentrating water into the 100 ml collection bottle (scooping the algal net in the center of the pond eight times across ca. 3 meters). Sediment samples were collected from random six locations from the bottom of the pond by collecting a total of ca. 450 g from the top 2 cm layer. Nine tree leaves (elm or oak) were collected per pond at random locations into the 50 ml sterile tubes. When the frog clutches emerged at the ponds at random location, jelly samples from approximately 30 eggs were collected for *R. dalmatina* by placing the samples into a sterile 50 ml tube, after photographing the clutch to determine developmental stage. The first sampling of clutches was performed on the 25th of March 2019 and the last on the 18th of April 2019 (Lelm 1 and Lelm2) and 11th of April 2019 (in Dahlum sampling site, i.e., no clutches in Dahlum on 18th of April 2019). Total number of collected samples was 100; 24 for water, sediment and leaves samples and 28 for clutch samples (Online Resource 1, Table S1). All samples, except clutches, were frozen at -20 °C (maximum of 2 h after collection) until further processing.

The algal colonization rate and embryo developmental stage were recorded for *R. dalmatina* clutch samples (Online Resource 1, Table S1; Fig. 1c-d). Embryo developmental stages were categorized to (i) egg to very early embryo, i.e., approximately corresponding to stages 1-14 according to the classification of Gosner (1960); (ii) early to moderately developed embryo, larval form not yet fully developed, approximate Gosner stages 15-19; (iii) hatchling with external gills but still inside egg, approximate Gosner stages 20-22; (iv) hatched embryo, approximate Gosner stages over 22. In a laboratory, clutch samples in 50 ml tubes were immediately poured onto Petri dishes, where ca. 4 ml of the clutches mass (without

embryos) were transferred (with sterile 2 ml syringes) to new sterile 50 ml tubes avoiding the obvious outer 'environmental contamination' as much as possible. About 20 ml of RNAlater solution was added on top of each 4 ml clutch sample, briefly vortexed, and stored at 4 ºC. Preparing clutch samples for DNA isolation process included vortexing the samples at maximum speed (using Vortex Genie 2) for 3 min, following centrifugation at 3,900 rpm for 10 min. After removing the supernatant, only the remaining 'greenish' pellet was subjected to DNA extraction. Water samples were thawed at room temperature, larger organic particles were removed by pouring the water through a 1 mm sieve (sieve was cleaned with 5% bleach and 70% EtOH after processing each sample). The remainder of each sample was centrifuged at 3,900 rpm for 10 min, followed by water removal by pipetting. Sediment samples were dried at 49 ºC for ca. 60 h, following grinding to a fine powder (in a sterile Whirl-Pak bag) to homogenize the sample. For the leaf samples, 20 ml of 96% EtOH was added and samples in 50 ml tubes were subjected to 3 min vortexing at maximum speed. Tubes were centrifuged at 3,900 rpm for 10 min, following EtOH removal by pipetting. For water and leaf samples, the remaining pellets (after supernatant removal) were used for DNA extraction (wet weight < 0.2 g). DNA was extracted from 0.1 g of dried and homogenized sediments.

Molecular analyses

All DNA extractions were performed using DNeasy PowerSoil Kit (Qiagen, Germany) following the manufacturer's instructions. PCRs were performed using uniquely tagged primers (8 bp + 2-4 bp heterogeneity spaces; Online Resource 1, Table S2) for amplifying fragments of RuBisCO large subunit (rbcL) and eukaryotic small subunit of ribosomal RNA (18S SSU rRNA). For amplifying the rbcL region, we used the newly designed primers, rbcL-646Fcl (5'-ATG CGT TGG MGW GAY CGT TTC-3') and rbcL-998Rcl (5'-GTT CHC CTT CWA RTT TWC CWA CWA C-3'), modified from Kelly et al. (2018; rbcL646F and rbcL998R, designed for diatoms) to amplify a wider range of photosynthetic micro-algae (targeting especially Chlamydomonadales). These new rbcL primers amplify a fragment of 333-336 bp. For amplifying 18S (V9 region), we used universal primers Euk1391f (5'-GTA CAC ACC GCC CGT C-3') and EukBr (5'-TGA TCC TTC TGC AGG TTC ACC TAC-3') (Amaral-Zettler et al. 2009; Stoeck et al. 2010). PCR mix, 25 µl per sample, consisted of 5 µl of Hot Start FirePol Master Mix (Solis BioDyne, Estonia), 0.5 µl forward and reverse primers (10 µM), 1 µl of template DNA, and 18 µl of nuclease-free water. PCR conditions for rbcL included initial hot-start at 95 ºC for 15 min, following 35 cycles of 95 ºC for 30 s, 55 ºC for 45 s, 72 ºC for 1 min, and final extension at 72 ºC for 10 min. PCR conditions for 18S included initial hot-start at 95 ºC for 15 min, following 35 cycles of 94 ºC for 45 s, 57 ºC for 1 min, 72 ºC for 1 min 90 s, and final extension at 72 ºC for 10 min. Two replicate PCRs were performed per sample. Sample replicates were pooled, and the yield of PCR products were checked via gel electrophoresis by pipetting 5 µl PCR product on 1% agarose gel. All PCR products were pooled as based on their relative

quantity (as observed on the gel) and purified using Favor-Prep™ Gel/PCR Purification Kit (Favorgen-Biotech Corp., Austria), following the manufacturer's instructions. Steps of DNA extraction, PCR, and sequencing included both negative and positive controls. Negative controls included blank DNA extractions and also PCRs with no-template DNA. DNA extracts from cultured species of diatoms were used as a positive control to monitor the functionality of PCRs and sequencing. Additionally, sixteen 'un-used tag' control samples were used (Online Resource 1, Table S2) to account for potential 'tag-switching' errors (Taberlet et al. 2018). All molecular procedures were performed under a laminar flow clean bench, with 30 min UV sterilization prior to and after each step. Sequencing was performed on an Illumina MiSeq instrument using MiSeq Reagent Kit v2 (2x250). Illumina sequencing data sets have been deposited in the Sequence Read Archive (SRA), BioProject ID: PRJNA714784.

Bioinformatics

Raw paired-end Illumina sequencing data were processed in the PipeCraft platform (Anslan et al. 2017), which included merging paired-end reads, quality filtering, chimera filtering, clustering, and formation of operational taxonomic units (OTUs) tables for both (rbcL and 18S) genes. Paired-end reads merging and quality filtering were processed using vsearch (Rognes et al. 2016); maximum expected error threshold of 1 (--fastq_maxee = 1) and discarding sequences with ambiguous bases (--fastq_maxns = 0). Putative chimeric reads were filtered using the uchime_denovo algorithm in vsearch (default settings). Few additional reads where a full-length primer string was detected inside the sequence (i.e., 'multiprimer artefacts') were discarded using PipeCraft built-in module ('remove primer artefacts'). Clustering of the sequences was performed using the UPARSE algorithm (Edgar 2013) with a 97% sequence similarity threshold. For taxonomy assignment, representative sequences (UPARSE centroids) for each OTU were compared against EMBL v142 (Kanz et al. 2005) reference database using blastn algorithm (Camacho et al. 2009). Based on the control samples and blastn results, the OTU tables were further checked and filtered to remove potential contaminants and mitigate tag-switching errors. Detailed OTU table curation is described in Online Resource 2 (extended methods).

OTU table curation

Based on the control samples, the resulting OTU tables were further checked and filtered to remove potential contaminants and mitigate tag-switching errors. First, tag-switching errors were corrected by calculating the relative abundance of sequences of each OTU per sample. The whole table was then filtered to include only sequences (for each OTU per sample) that had higher relative read abundances compared with average sequence abundance in the 'un-used tag' control samples. The average sequence abundance (per OTU in a sample) in the 'un-used tag' control samples was 0.00421 and 0.0060 for rbcL and 18S OTU

tables, respectively, summing up to 10 sequences per control sample (maximum count per OTU was 2 reads). Further, ≤ 2 read occurrences per OTU per samples were discarded from the whole data sets. Based on negative extraction and negative PCR control samples, an OTU was considered contamination when the sequence count for the corresponding OTU in the negative control was higher compared with biological samples. No contaminant OTUs were detected from rbcL data, but several potential contaminants (mostly taxa from Protista and Stramenopila) were detected in the data generated with universal 18S primers based on their presence in negative controls and thus removed. OTUs assigned to metazoans, bacteria, land plants, soil fungi, bryophytes, lichens and OTUs that did not get a blastn match were removed from the analyses. Additionally, OTUs with < 80% of sequence similarity and < 85% sequence coverage against reference read in the EMBL database were marked as unassigned and removed from the analyses.

Statistics

Permutational Analysis of Variance (PERMANOVA, with 9999 permutations) was used for detecting the effects of substrate (water, leaves, sediments, clutches), sampling site (Lelm1, Lelm2, Dahlum), and sampling date (8 sampling dates) on the OTU community composition using PRIMER v6 (Clarke and Gorley 2006). Log-transformed Bray-Curtis as well as UniFrac distance (unweighted) OTU matrices were used for the PERMANOVA analyses. UniFrac distances were calculated using the PhyloMeasures package (Tsirogiannis and Sandel 2016) in R (R-Core-Team 2019) using Maximum-likelihood based phylogenetic rbcL and 18S amplicon trees generated with RAxML (Stamatakis 2014) under the GTRGAMMA model. Because sequencing depth may affect the OTU abundance (thus community composition patterns), sequence counts per sample were used as a covariate (Type I SS). Obvious outliers were screened with non-metric multidimensional scaling (NMDS) analyses and removed prior to PERMANOVA. Distance-based linear model (DistLM) with forward selection procedure and AICc selection criterion (using PRIMER v6) was used to detect the most important factors affecting the algal (rbcL data) and micro-eukaryotic (18S data) communities associated with frog clutch samples (9999 permutations). For identifying OTUs that are consistently present in a given substrate type (i.e., indicator OTUs), indicator species analyses were performed using the 'indicspecies' library (De Caceres et al. 2016) in R. Interactive visualization graphs for indicator OTUs (taxa) were generated using Krona chart (Ondov et al. 2011). Bar plots for taxonomic distributions were generated using 'phyloseq' package (McMurdie and Holmes 2013) in R. Temporal distance decay of similarity of OTU composition were explored by Mantel tests using the 'vegan' package (Oksanen et al. 2015) in R.

Culturing, Sanger sequencing and RNAseq

Single *R. dalmatina* egg envelopes were cut open with microsurgery scissors, and algal cells were extracted with a micromanipulator (Patchman NP2, Eppendorf, Germany). The obtained algal cells were cultivated in Waris H medium (McFadden and Melkonian 1986) under standard conditions (light/dark 14:10 hours at 16 ºC and 5000 K provided by LED daylight strips, SunLike Linear Z 560-52, Lumitronix, Germany). For DNA extraction, the culture was centrifuged at 4,000 x g for 20 min at 4 °C. The pellet was re-suspended in 700 µl Genomic Lysis Buffer using the Quick DNA prep kit (Zymo Research, US) following manufacturer's protocol for cell suspensions. The complete 18S rDNA was amplified in PCR reactions using 1.5 µl genomic DNA template, 12.5 µl Red Taq Polymerase Master Mix (VWR Chemicals International, Belgium), and each 2.5 µl 18SFor 5'-AAC CTG GTT GAT CCT GCC AGT-3' and 18S-Rev 5'-TGA TCC TTC CGC AGG TTC ACC TAC-3' primer (Medlin et al. 1988). The thermal amplification program followed Schoenle et al. (2019): initial denaturation at 98 ºC for 2 min, followed by 35 cycles of 30 s at 98 ºC, 45 s at 55 ºC and 2 min 30 s for 72 ºC, and ending with a final elongation step of 72 ºC for 10 min. PCR products were purified using the PCR purification kit (Jena Bioscience, Germany) following the manufacturer's protocol and sequenced with the corresponding amplification primers at GATC Biotech Cologne. Sequence editing and quality check were performed using Bioedit Sequence Alignment Editor (v7.2.6; Hall 1999). The 18S sequence (isolate MVRNA93) has been deposited in Genbank (Benson et al. 2013), under accession number MW723501.

For RNA extraction, a sample of ca. 100 mg of the culture was preserved in RNAlater at -80°C. Prior to extraction, the sample was homogenized in 2 ml tube, containing 1 ml of trizol, with steal beads using the tissue lyser Precellys (speed 6000, 2x 55 sec in between 10 sec break). The samples were centrifuged for 10 min at 12,000 g at 4 °C. The solution was transferred to a new tube and incubated at room temperature (RT) for 5 min, after which 200 µl chloroform was added, samples were vortexed for 15 s, incubated for 3 min at RT and centrifuged for 15 min at 12,000 g at 4 °C. The upper phase was transferred to a new tube and 500 µl isopropanol was added. The solution was incubated for 10 min at RT and centrifuged for 15 min at 12,000 g at 4 °C. The supernatant was removed, 1 ml 75% EtOH was added to the pellet (using DEPC water) and the sample was vortexed until the pellet was resuspended. The samples were centrifuged for 5 min at 7,500 g at 4 °C and the supernatant was removed. The pellet was dried and dissolved in DEPC water. The RNA was precipitated by adding 200 µl 5M LiCl solution and incubated for 1.5 h at -20 °C. After incubation, the solution was centrifuged for 20 min at 14,000 x g at 4 °C. The supernatant was removed, the pellet washed with 500 µl 75% EtOH and then centrifuged for 10 min at 14,000 x g at 4 °C. The pellet was again dried and dissolved in DEPC water. Sequencing was carried out with a High Output 2x150 cycle kit on an Illumina NextSeq instrument. Reads were quality-trimmed and filtered using Trimmomatic v. 0.32 (Bolger et al. 2014) with default settings (i.e. slidingwindow: 4:5,

leading: 5, trailing: 5, minlength: 25). Filtered reads were used for *de novo* transcriptome assembly using Trinity v. 2.1.0 (Grabherr et al. 2011) following a published protocol (Haas et al. 2013). Illumina NextSeq sequencing data is deposited in the Sequence Read Archive (SRA), BioProject ID: PRJNA712983.

Phylogenetics

For phylogenetic analyses, we relied on sequences of the 18S rRNA gene because most previous studies focusing on *Oophila* used this gene, and numerous comparative sequences are therefore available. Analyses were performed at levels of taxonomy and sequence length to make the best use of all available data, and considering that fully combining all sequences is not feasible due to extremely different sequence lengths and sequence variation. Furthermore, we compiled multi-gene datasets from transcriptomic data containing 18 nuclear protein-coding genes. All alignments have been uploaded to Figshare (doi: 10.6084/m9.figshare.14216588).

Dataset 1. To understand the identity and overall placement of the various algal isolates sequenced from amphibian clutches, we assembled a data set of all 18S rRNA sequences of such algae from Genbank (Benson et al. 2013), plus a comparative selection of Chlorophyta comprising (i) all outgroups and comparative sequences used in previous publications (Correia et al. 2020; Kim et al. 2014; Muto et al. 2017; Nema et al. 2019); (ii) all sequences assigned to *Oophila* in these studies and otherwise available from Genbank; (iii) all sequences with >98% sequence identity and >80% sequence coverage obtained via BLAST searches in the Genbank nucleotide collection, using the most complete 18S sequences of *Oophila* "Clade A" and "Clade B" as queries, and (iv) a selection of sequences obtained via metabarcoding, chosen as follows: we first selected all OTUs with at least 10 reads from *Rana dalmatina* clutch samples, and added all other OTUs present with fewer reads in the clutches and matching *Chlorococcum* or *Oophila* in the SILVA database. We then added these sequences to our 18S alignment, ran a preliminary Maximum Likelihood (ML) analysis in MEGA7 (Kumar et al. 2016), and removed all OTUs not clustering (phylogenetically) within Chlorophyta. Eventually, we also aligned all OTU sequences (from clutches and other microhabitats) with *Oophila* representative sequences for "Clade A" and "Clade B" and thereby extracted from the metabarcoding data one additional OTU sequence that matched the Sanger sequence of our isolate (MVRNA93). All sequences in this dataset were aligned in MAFFT (Katoh et al. 2005) with the G-INS-i (accurate) option. Because of the very short length of the metabarcoding sequences, a reliable phylogenetic reconstruction was not possible including all of them; we therefore excluded all metabarcoding sequences except two (the ones best matching *Oophila* "Clade A" and "Clade B"), and performed a ML analysis of the resulting data set (alignment length 1878 bp) in RaxML (Stamatakis 2014) with a GTR+G model.

Dataset 2. To visualize the placement of additional Chlorophyta OTUs found by metabarcoding in different parts the green algae tree, we used the initial full alignment from Dataset 1 but trimmed the 18S sequences to 139 bp to match the metabarcoding fragment. Shorter sequences were excluded. The resulting data set was submitted to ML analysis in RaxML. For metabarcoding OTUs, only those found in *Rana dalmatina* clutch samples and corresponding to green algae were included in this data set.

Dataset 3. To better visualize the variation of sequences assigned to *Oophila* "Clade A" and "Clade B" relative to other related algae, we used the 18S sequences from Dataset 1 as a basis, and selected those taxa that either (i) were classified as *Oophila*, (ii) were classified as *Chlorococcum* and by Dataset 1 analysis were placed in a clade with sequences classified as *Oophila*, and (iii) were nested within the *Oophila* and *Chlorococcum* clades, or were direct sister taxa to samples classified as *Oophila*. These sequences were then realigned with MAFFT, and the alignment (1878 bp) submitted to an analysis with RaxML.

Datasets 4 and 5. To obtain confirmation of algae's placement isolated from amphibian clutches in two very distinct branches of the Chlorophyta from a genome-wide selection of markers, we used a recently published phylotranscriptomic data set across green plants (Leebens-Mack et al. 2019). For Dataset 4, we downloaded the nucleotide alignments of 386 single-copy nuclear genes used in the study by Leebens-Mack et al. (2019), and kept sequences of 115 Chlorophyta, plus four Streptophyta as outgroups. We then used the newly obtained transcriptome assembly from an algal isolate cultured from German clutches of *Rana dalmatina* (MVRNA93), and an assembly of North American *Oophila* (reads available from SRA under SRR5445904) from a cultured isolate from the work of Burns et al. (2017). These two transcriptomes corresponded to *Oophila* "Clade A" and "Clade B" of Nema et al. (2019), respectively. Each contig was blastn searched against all sequences of the original phylotranscriptomic data set and hits with >80% identity were considered as matches. We selected 18 genes (Online Resource 1, Table S8) with matches in both transcriptomes, added the respective new transcriptome sequences to the original alignments, and performed codon-based Clustal W alignments in MEGA7 (Kumar et al. 2016). After initial exploratory phylogenetic analyses at the nucleotide level, the alignments were translated to amino acids, yielding a total alignment length of 6758 amino acid (aa) positions for 121 taxa. GBLOCKS (Castresana 2000) was then used to remove unalignable portions, with a minimum block length of 2 positions, and allowing gaps in 50% of sequences. We then performed a ML analysis of the concatenated alignment in RaxML under the GTR + 4Γmodel including a search of the best tree and 100 bootstrap replicates. To improve the analytical power for relationships within or target group (by including additional positions that were difficult to align for more distantly related algae), we ran a second analysis with the same settings for a reduced dataset (Dataset 5) with all taxa from a well-supported clade containing all taxa from Dataset 4 belonging to Chlamydomonadales, Sphaeropleales, Chaetophorales, Oedogoniales, and Chaetopeltidales, as well as representative outgroups from various other Chlorophyta clades (a total of 71 taxa for 6032 aa positions

before GBLOCKS). The final alignments after GBLOCKS contained 4219 aa (Dataset 4) and 4892 aa (Dataset 5).

Dataset 6. We also assessed the phylogenetic placement of amphibian clutch-associated algae using sequences of the rbcL marker. For this, we first extracted rbcL sequences from the two transcriptomes (MVRNA93 and SRR5445904), then retrieved all rbcL sequences of *Oophila* and *Chlorococcum* from Genbank, plus sequences matching our transcriptome rbcL sequences with 87% (SRR5445904) and 90% (MVRNA93) identity. To these sequences we added the metabarcoding rbcL fragments of *Oophila* "Clade B" plus a series of additional algae clearly and in high read numbers associated to *Rana dalmatina* clutches.

References:

Amaral-Zettler LA, McCliment EA, Ducklow HW, Huse SM (2009) A method for studying protistan diversity using massively parallel sequencing of V9 hypervariable regions of small-subunit ribosomal RNA genes. PLoS ONE 4:e6372

Anslan S, Bahram M, Hiiesalu I, Tedersoo L (2017) PipeCraft: flexible open-source toolkit for bioinformatics analysis of custom high-throughput amplicon sequencing data. Mol Ecol Resour 17:e234-e240

Benson DA, Cavanaugh M, Clark K, Karsch-Mizrachi I, Lipman DJ, Ostell J, Sayers EW (2013) GenBank. Nucleic Acids Res 41:D36-D42

Bolger AM, Lohse M, Usadel B (2014) Trimmomatic: a flexible trimmer for Illumina sequence data. Bioinformatics 30:2114-2120

Burns JA, Zhang H, Hill E, Kim E, Kerney R (2017) Transcriptome analysis illuminates the nature of the intracellular interaction in a vertebrate-algal symbiosis. Elife 6:e22054

Camacho C, Coulouris G, Avagyan V, Ma N, Papadopoulos J, Bealer K, Madden TL (2009) BLAST+: architecture and applications. BMC Bioinformatics 10:421

Castresana J (2000) Selection of conserved blocks from multiple alignments for their use in phylogenetic analysis. Mol Biol Evol 17:540-552

Clarke K, Gorley R (2006) PRIMER V6: User manual/tutorial. Primer-E Ltd Plymouth, 192pp

Correia N, Pereira H, Silva JT, Santos T, Soares M, Sousa CB, Schüler LM, Costa M, Varela J, Pereira L (2020) Isolation, identification and biotechnological applications of a novel, robust, free-living *Chlorococcum* (*Oophila*) *amblystomatis* strain isolated from a local pond. Applied Sciences 10:3040

De Caceres M, Jansen F, De Caceres MM (2016) Package 'indicspecies'.

Edgar RC (2013) UPARSE: highly accurate OTU sequences from microbial amplicon reads. Nat Methods 10:996-998

Gosner KL (1960) A simplified table for staging anuran embryos and larvae with notes on identification. Herpetologica 16:183-190

Grabherr MG, Haas BJ, Yassour M, Levin JZ, Thompson DA, Amit I, Adiconis X, Fan L, Raychowdhury R, Zeng Q (2011) Trinity: reconstructing a full-length transcriptome without a genome from RNA-Seq data. Nat Biotechnol 29:644-652

Haas BJ, Papanicolaou A, Yassour M, Grabherr M, Blood PD, Bowden J, Couger MB, Eccles D, Li B, Lieber M (2013) *De novo* transcript sequence reconstruction from RNA-seq using the Trinity platform for reference generation and analysis. Nature protocols 8:1494-1512

Hall TA BioEdit: a user-friendly biological sequence alignment editor and analysis program for Windows 95/98/NT. In: Nucleic acids symposium series, 1999. vol 41. [London]: Information Retrieval Ltd., c1979-c2000., pp 95-98

Kanz C, Aldebert P, Althorpe N, Baker W, Baldwin A, Bates K, Browne P, van den Broek A, Castro M, Cochrane G (2005) The EMBL nucleotide sequence database. Nucleic Acids Res 33:D29-D33

Katoh K, Kuma K-i, Toh H, Miyata T (2005) MAFFT version 5: improvement in accuracy of multiple sequence alignment. Nucleic Acids Res 33:511-518

Kelly M, Boonham N, Juggins S, Killie P, Mann D, Pass D, Sapp M, Sato S, Glover R (2018) A DNA based diatom metabarcoding approach for Water Framework Directive classification of rivers. Bristol: Environment Agency

Kim E, Lin Y, Kerney R, Blumenberg L, Bishop C (2014) Phylogenetic analysis of algal symbionts associated with four North American amphibian egg masses. PLoS ONE 9:e108915

Kumar S, Stecher G, Tamura K (2016) MEGA7: Molecular Evolutionary Genetics Analysis Version 7.0 for Bigger Datasets. Mol Biol Evol 33:1870-1874

Leebens-Mack JH et al. (2019) One thousand plant transcriptomes and the phylogenomics of green plants. Nature 574:679-685

McFadden G, Melkonian M (1986) Use of Hepes buffer for microalgal culture media and fixation for electron microscopy. Phycologia 25:551-557

McMurdie PJ, Holmes S (2013) phyloseq: an R package for reproducible interactive analysis and graphics of microbiome census data. PLoS ONE 8:e61217

Medlin L, Elwood HJ, Stickel S, Sogin ML (1988) The characterization of enzymatically amplified eukaryotic 16S-like rRNA-coding regions. Gene 71:491-499

Muto K, Nishikawa K, Kamikawa R, Miyashita H (2017) Symbiotic green algae in eggs of *Hynobius nigrescens*, an amphibian endemic to Japan. Phycol Res 65:171-174

Nema M, Hanson ML, Müller KM (2019) Phylogeny of the egg-loving green alga *Oophila amblystomatis* (Chlamydomonadales) and its response to the herbicides atrazine and 2,4-D. Symbiosis 77:23-39

Oksanen J, Blanchet FG, Kindt R, Legendre P, Minchin PR, O'hara R, Simpson GL, Solymos P, Stevens M, Wagner H (2015) R package 'vegan': community ecology package.

Ondov BD, Bergman NH, Phillippy AM (2011) Interactive metagenomic visualization in a Web browser. BMC Bioinformatics 12:385

R-Core-Team (2019) R: A language and environment for statistical computing. R Foundation for Statistical Computing, Vienna, Austria. URL https://www.R-project.org/.

Rognes T, Flouri T, Nichols B, Quince C, Mahé F (2016) VSEARCH: a versatile open source tool for metagenomics. PeerJ 4:e2584

Schoenle A, Živaljić S, Prausse D, Voß J, Jakobsen K, Arndt H (2019) New phagotrophic euglenids from deep sea and surface waters of the Atlantic Ocean (*Keelungia nitschei*, *Petalomonas acorensis*, *Ploeotia costaversata*). Eur J Protistol 69:102-116

Stamatakis A (2014) RAxML version 8: a tool for phylogenetic analysis and post-analysis of large phylogenies. Bioinformatics 30:1312-1313

Stoeck T, Bass D, Nebel M, Christen R, Jones MD, BREINER HW, Richards TA (2010) Multiple marker parallel tag environmental DNA sequencing reveals a highly complex eukaryotic community in marine anoxic water. Mol Ecol 19:21-31

Taberlet P, Bonin A, Coissac E, Zinger L (2018) Environmental DNA: For biodiversity research and monitoring. Oxford University Press,

Tsirogiannis C, Sandel B (2016) PhyloMeasures: a package for computing phylogenetic biodiversity measures and their statistical moments. Ecography 39:709-714
