# Supplementary material for: Diversity and substrate-specificity of green algae and other micro-eukaryotes colonizing amphibian clutches in Germany, revealed by DNA metabarcoding
Source: Naturwissenschaften. 2021 Jun 28;108(4):29. doi: 10.1007/s00114-021-01734-0 (PMC8238718; doi:10.1007/s00114-021-01734-0)
Supplement: Supplementary file 4 — Supplementary file4 (PDF 4184 KB) [file 114_2021_1734_MOESM4_ESM.pdf]

Diversity and substrate-specificity of green algae and other micro-eukaryotes colonizing amphibian clutches in Germany, revealed by DNA metabarcoding

Sten Anslan[#], Maria Sachs, Lois Rancilhac, Henner Brinkmann, Jörn Petersen, Sven Künzel, Anja Schwarz, Hartmut Arndt, Ryan Kerney, Miguel Vences

#Corresponding author: s.anslan@tu-braunschweig.de; Zoological Institute, Technische Universität Braunschweig, Braunschweig, Germany

Journal: The Science of Nature

Online Resource 4. Supplementary Figures:

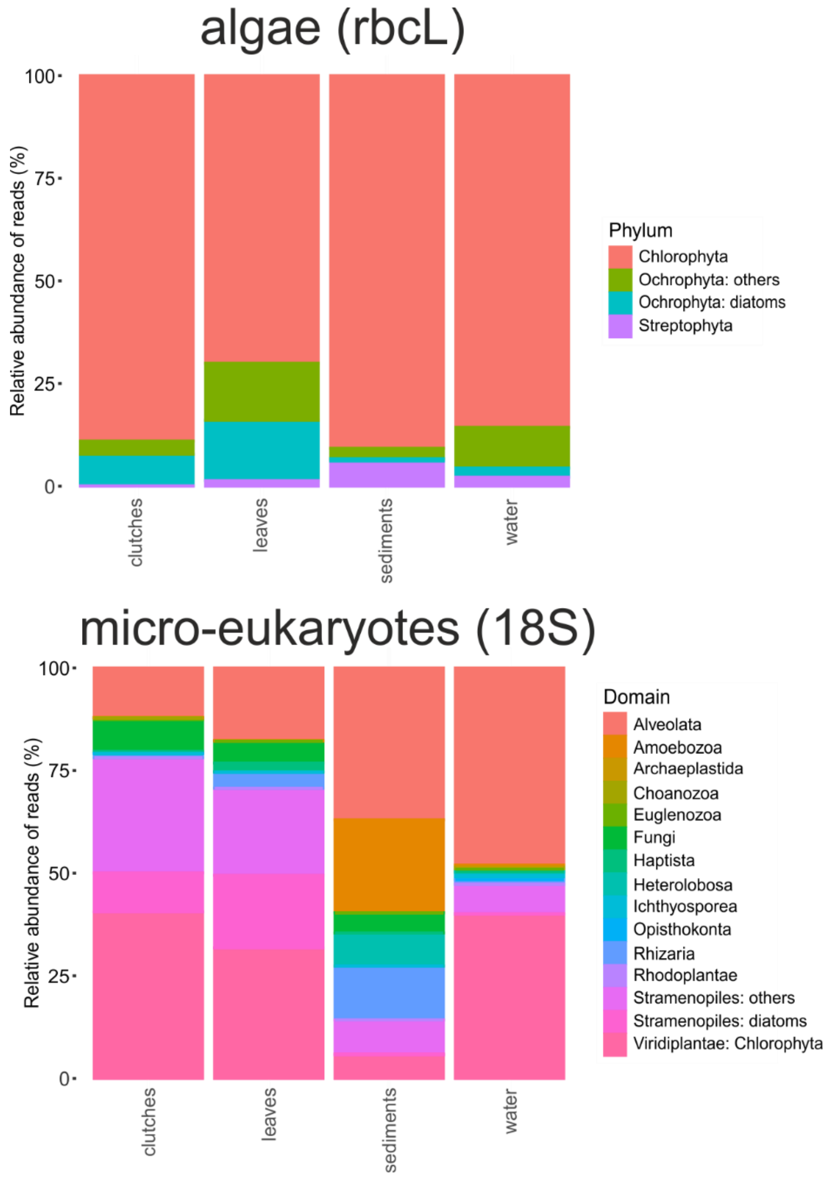

Figure S1. Relative abundance of rbcL and 18S reads from taxa associated with clutch, leaves, sediments and water samples. Phylum level distribution for rbcL, but Domain level distribution for 18S data, pointing out the Chlorophyta and diatoms groups.

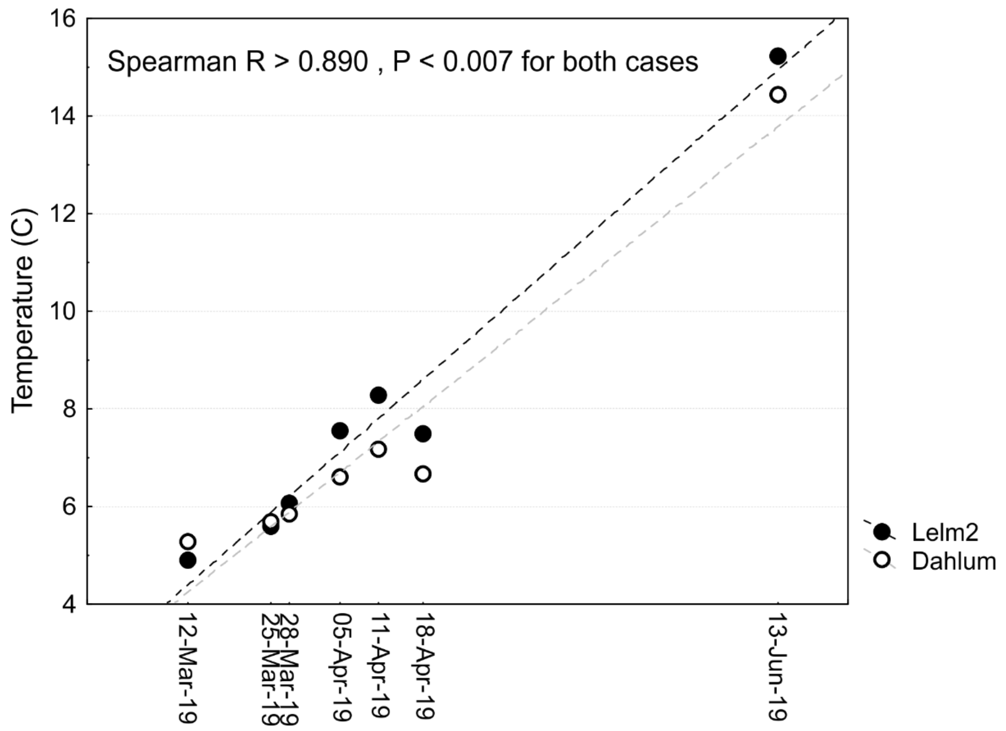

Figure S2. Correlation plot between sampling date and water temperature in ponds. Water temperature represents the average temperature across 10 days before (and including) the sampling date.

Figure S3. Interactive chart of indicator algal OTUs (taxa; rbcL marker) in *Rana dalmatina* clutches samples across three sampling sites. Percentage in the parentheses after indicator OTU represent the blastn identity percentage match for that taxon. Chart available for download at figshare.com, doi: **10.6084/m9.figshare.14229182**.

Figure S4. Interactive chart of indicator micro-eukaryotic OTUs (taxa; 18S marker) in *Rana dalmatina* clutches samples across three sampling sites. Percentage in the parentheses after indicator OTU represent the blastn identity percentage match for that taxon. Chart available for download at figshare.com, doi: **10.6084/m9.figshare.14229179**.

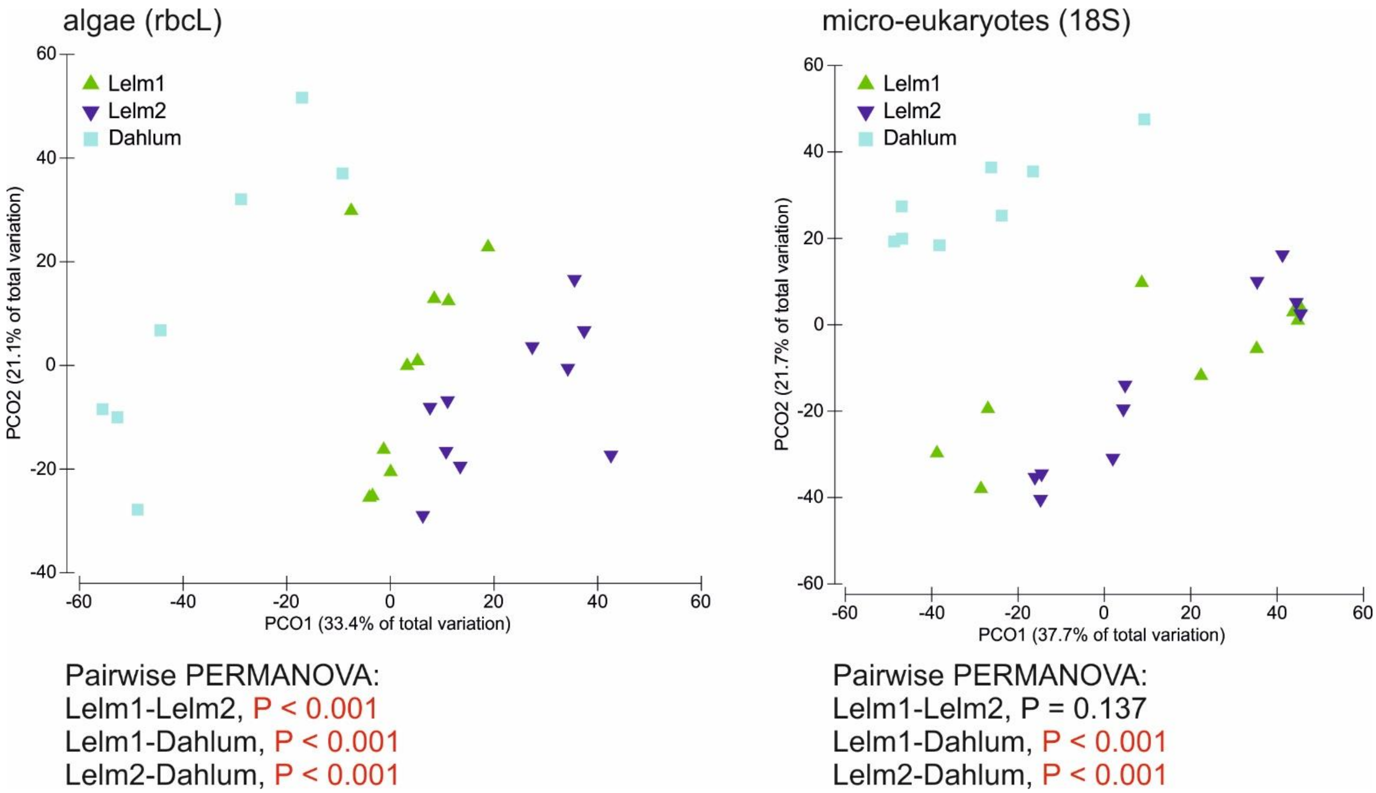

Figure S5. PCO plots for indicator OTUs data in *Rana dalmatina* clutch samples that demonstrate the clutch-associated community differences between sampling sites.

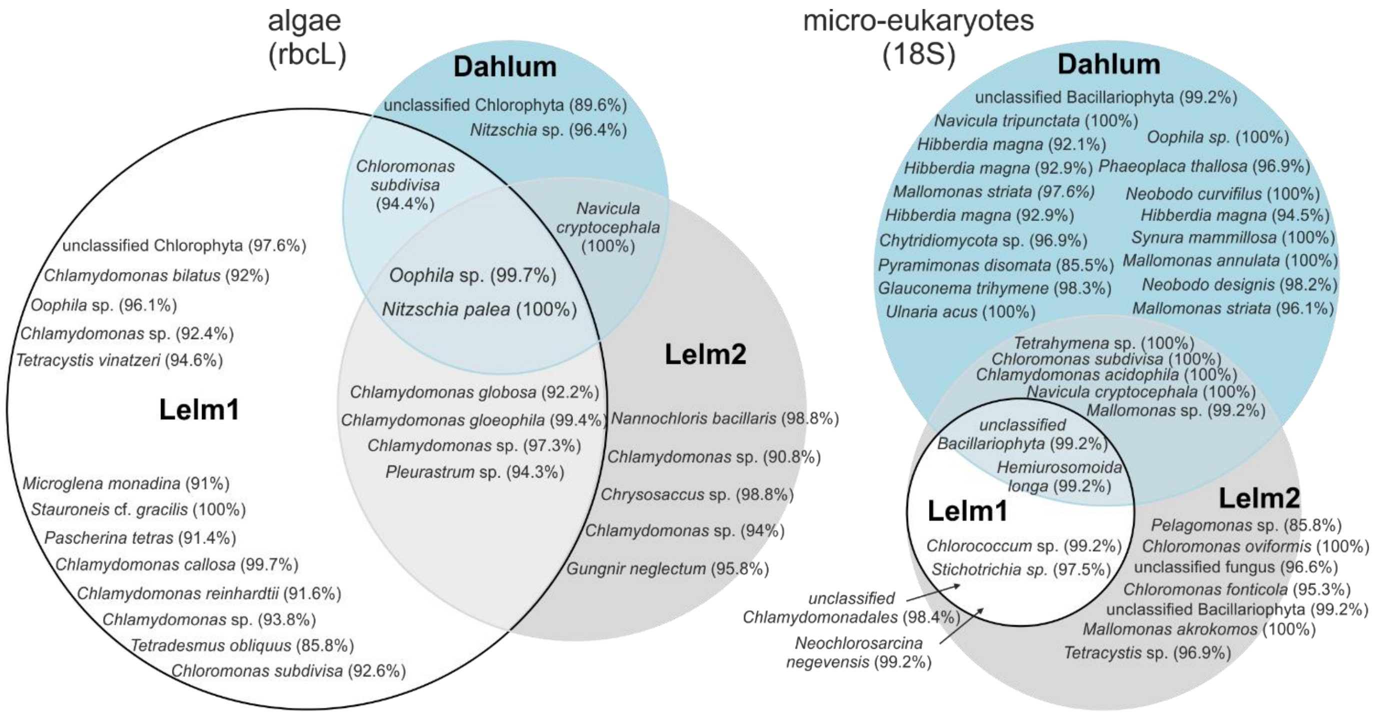

Figure S6. Venn diagrams for indicator OTUs in *Rana dalmatina* clutch samples separated by sampling site. Overlapping areas denote shared OTUs (i.e. OTUs that were determined as indicators for corresponding sites). Non-overlapping areas denote indicator OTUs that are unique to the corresponding sampling site. Note that the marked taxa on diagrams represent blastn first match where the percentage in between parentheses denote the blastn identity percentage. Total number of combined (Lelm1 + Lelm2 + Dahlum) indicator OTUs in clutch samples are 29 and 36 for rbcL and 18S data, respectively.

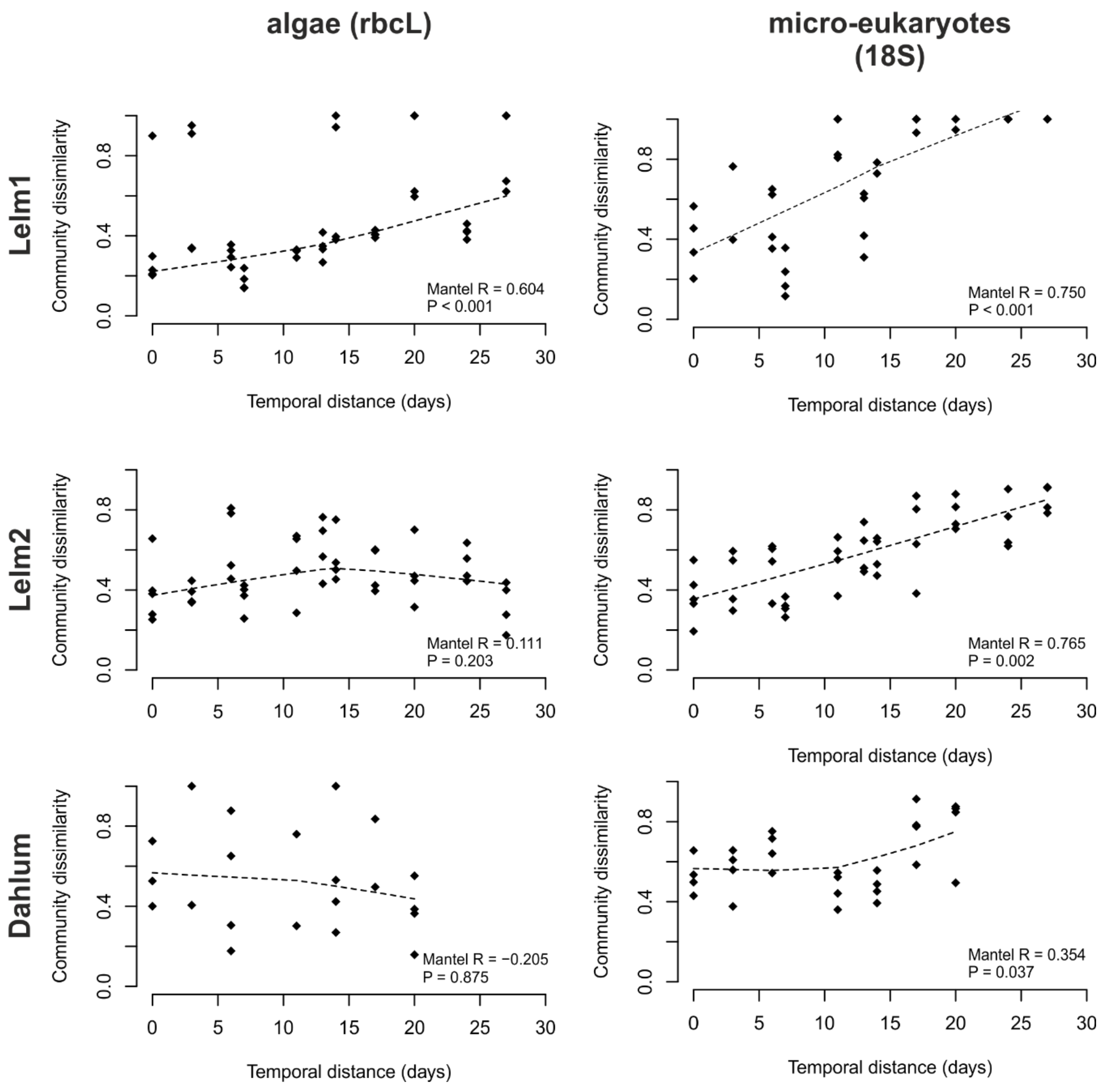

Figure S7. Relationships between indicator OTU community dissimilarity in *Rana dalmatina* clutch samples and temporal distance for each sampling site. Indicator OTUs per site include all identified indicators for clutch samples in the corresponding site (as in Online Resource 3).

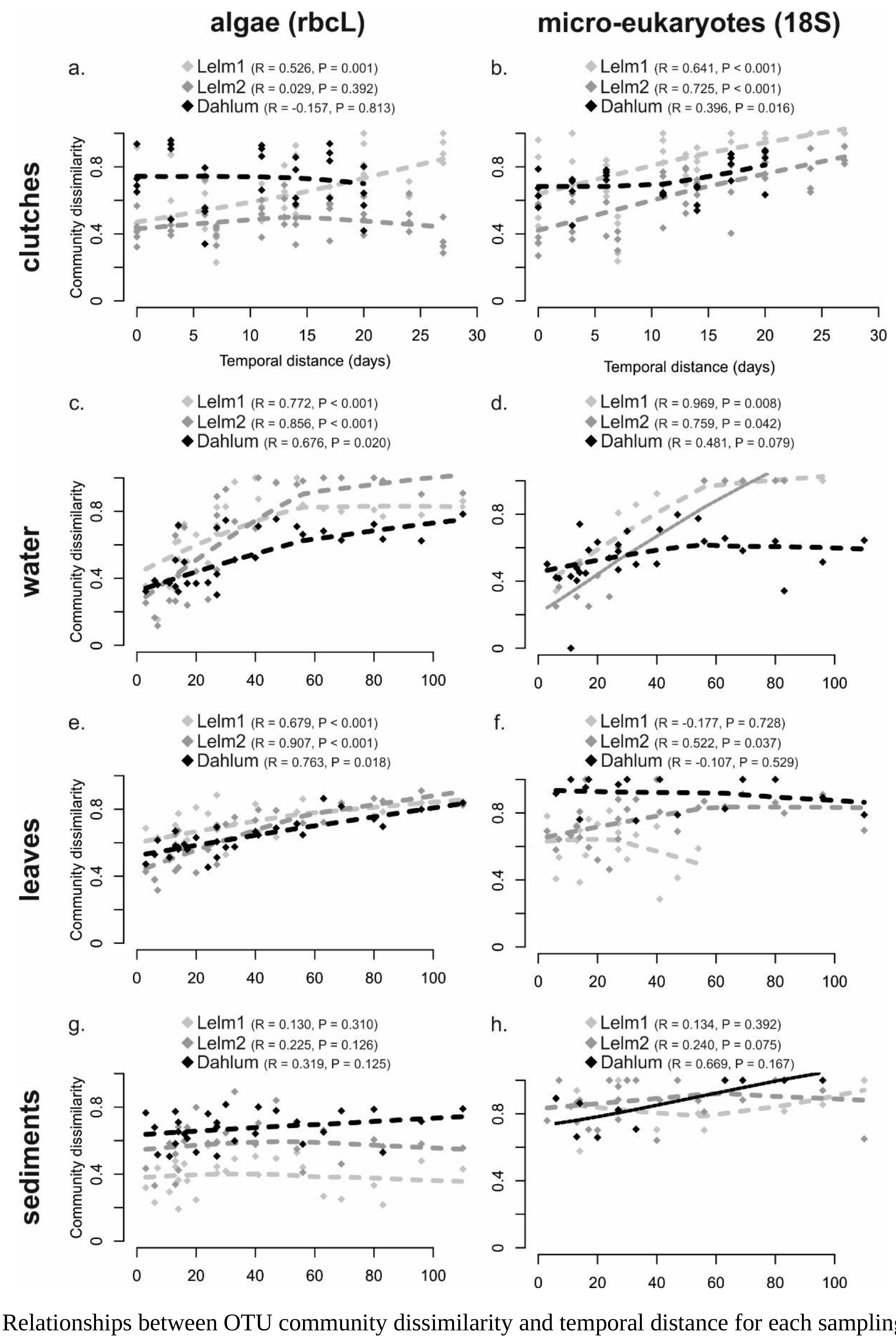

Figure S8. Relationships between OTU community dissimilarity and temporal distance for each sampling site: (a, b) Rana dalmatina clutch samples; (c, d) pond water surface samples; (e, f) leaf samples from the bottom of the pond; (g, h) pond sediment samples for rbcL and 18S data, respectively.

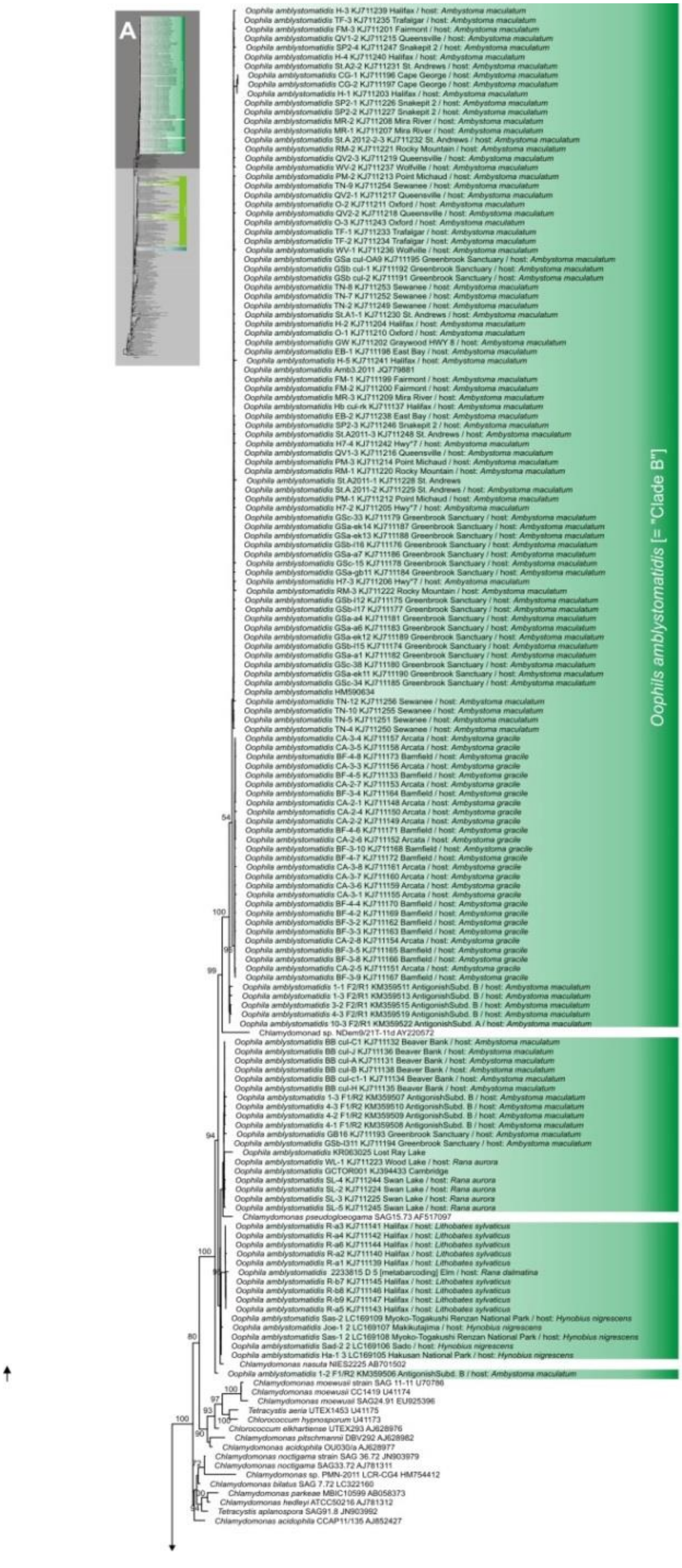

A
Oophila amblystomatidis [= "Clade B"]

Figure S9. Maximum Likelihood tree inferred from the full dataset of from DNA sequences of the 18S rRNA gene (Dataset 1; 1878 bp) comprising all sequences assigned to *Oophila* and related taxa, plus all outgroup taxa used, in previous studies and the new sequences obtained herein. Dark green marks the clade of amphibian-associated *Oophila* ("Clade B" according to Nema et al. 2019), light green marks the *Oophila*-like lineages found associated to amphibians within the *Chlorococcum* clade ("Clade A" according to Nema et al. 2019), and bluish green marks samples of amphibian-associated *Chlamydomonas gloeophila*. Numbers at nodes show bootstrap values in percent (only shown if >50% and removed from the shallowest nodes for better graphical representation).

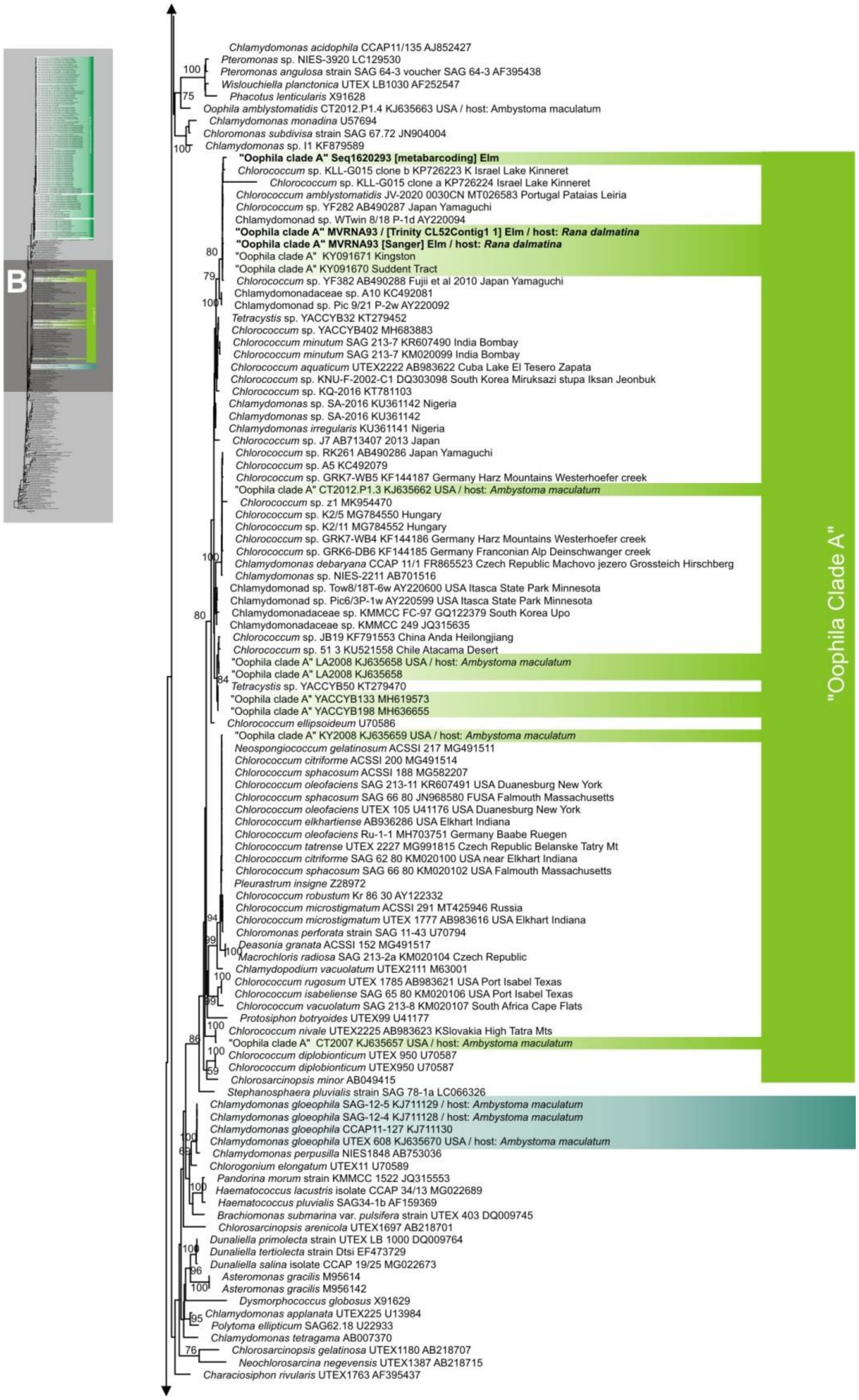

Figure S9. Continued.

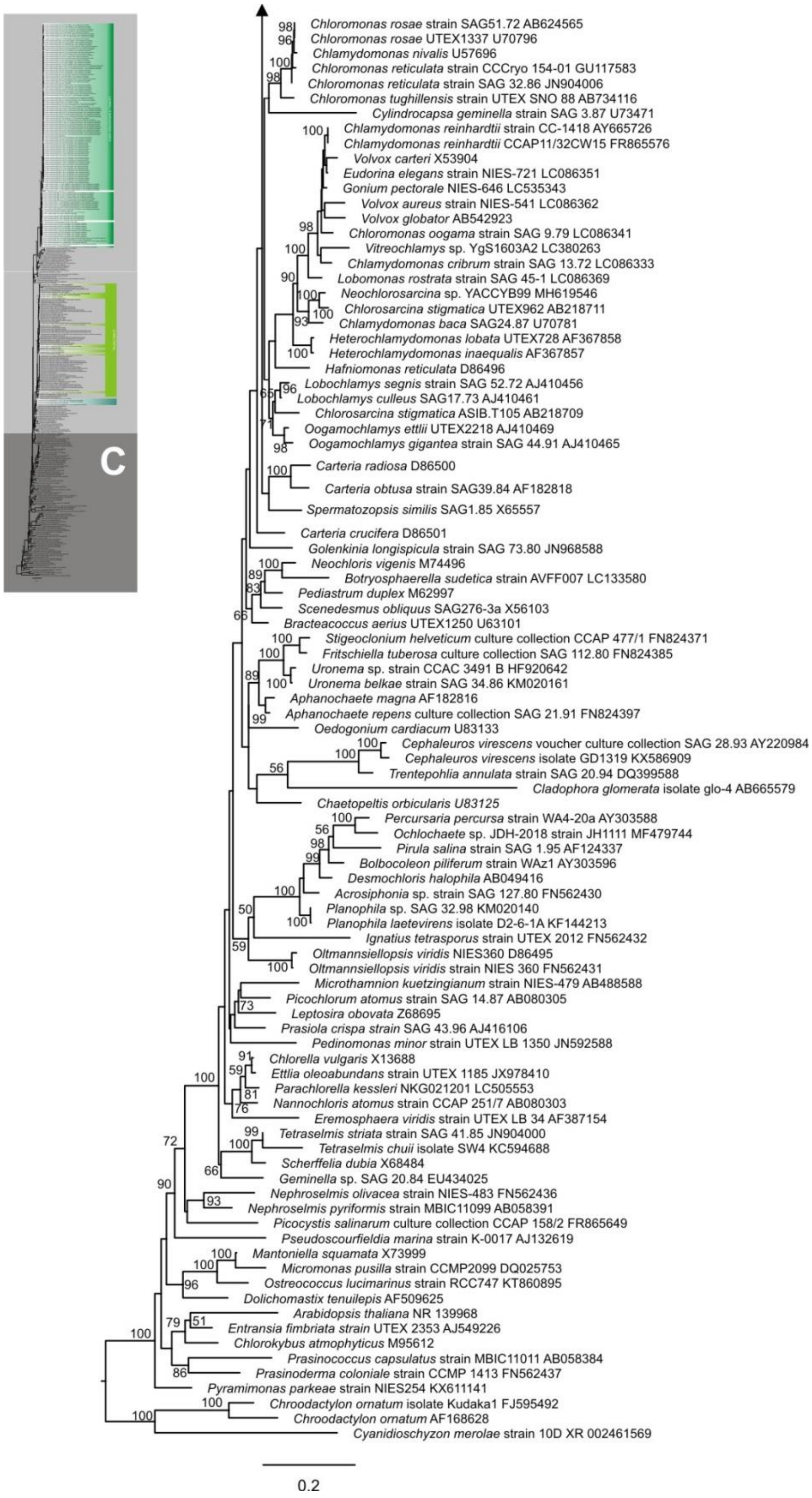

Figure S9. Continued.

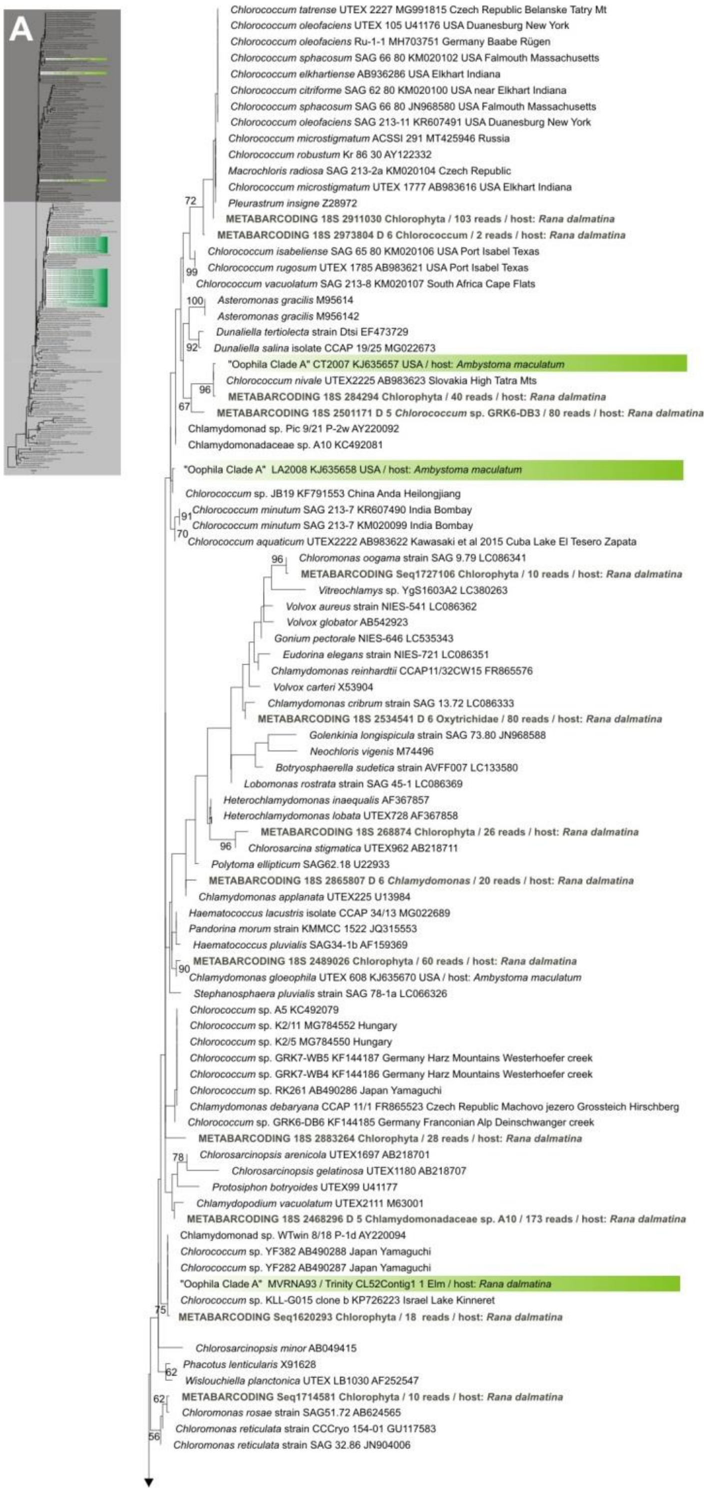

Figure S10. Maximum Likelihood tree inferred from Dataset 2, i.e., a subset of from DNA sequences of the 18S rRNA gene from Dataset 1 trimmed to 139 bp, plus metabarcoding-derived OTU Chlorophyta consensus sequences from ponds in the Elm, Germany, that were found associated to clutches of *Rana dalmatina*. Dark green marks the clade of amphibian-associated *Oophila* ("Clade B" according to Nema et al. 2019), light green marks the *Oophila*-like lineages found associated to amphibians within the *Chlorococcum* clade ("Clade A" according to Nema et al. 2019). Numbers at nodes show bootstrap values in percent (only shown if >50%). Note that samples from the metabarcoding study are found in numerous disparate phylogenetic positions, suggesting that many green algae occasionally and opportunistically can colonize amphibian clutches. Labels of metabarcoding sequences contain the total number of reads identified in clutch samples.

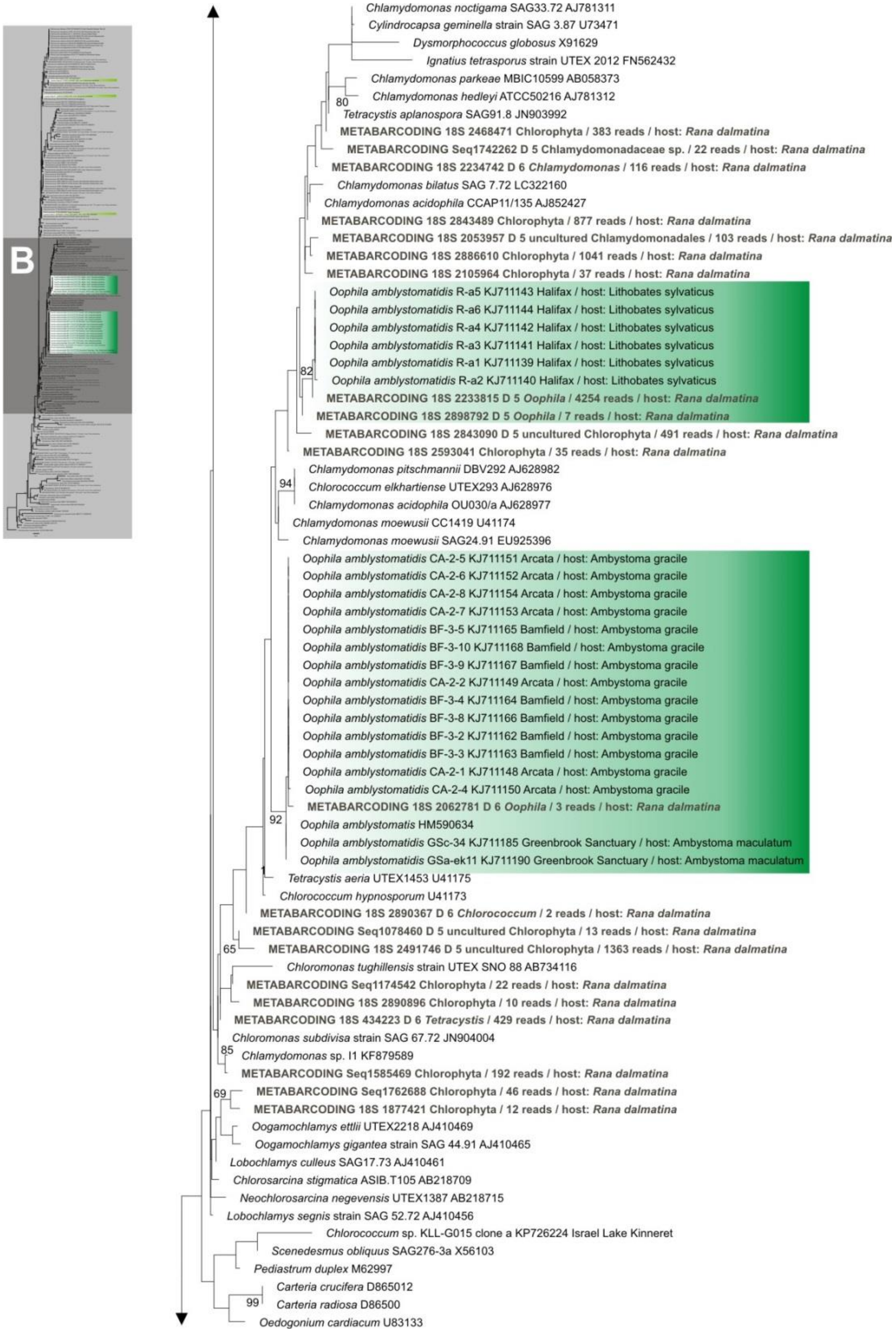

Figure S10. Continued.

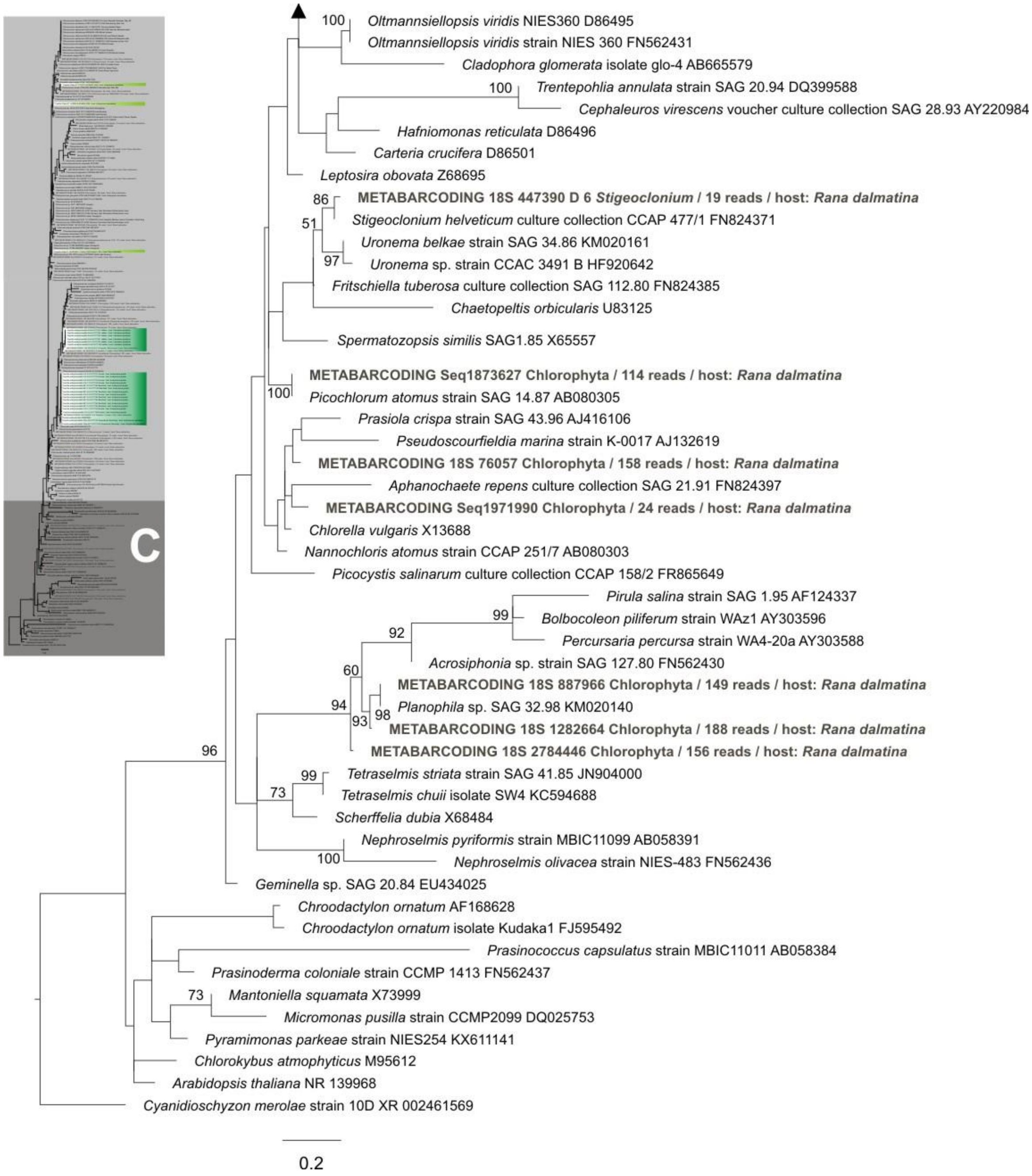

Figure S10. Continued.

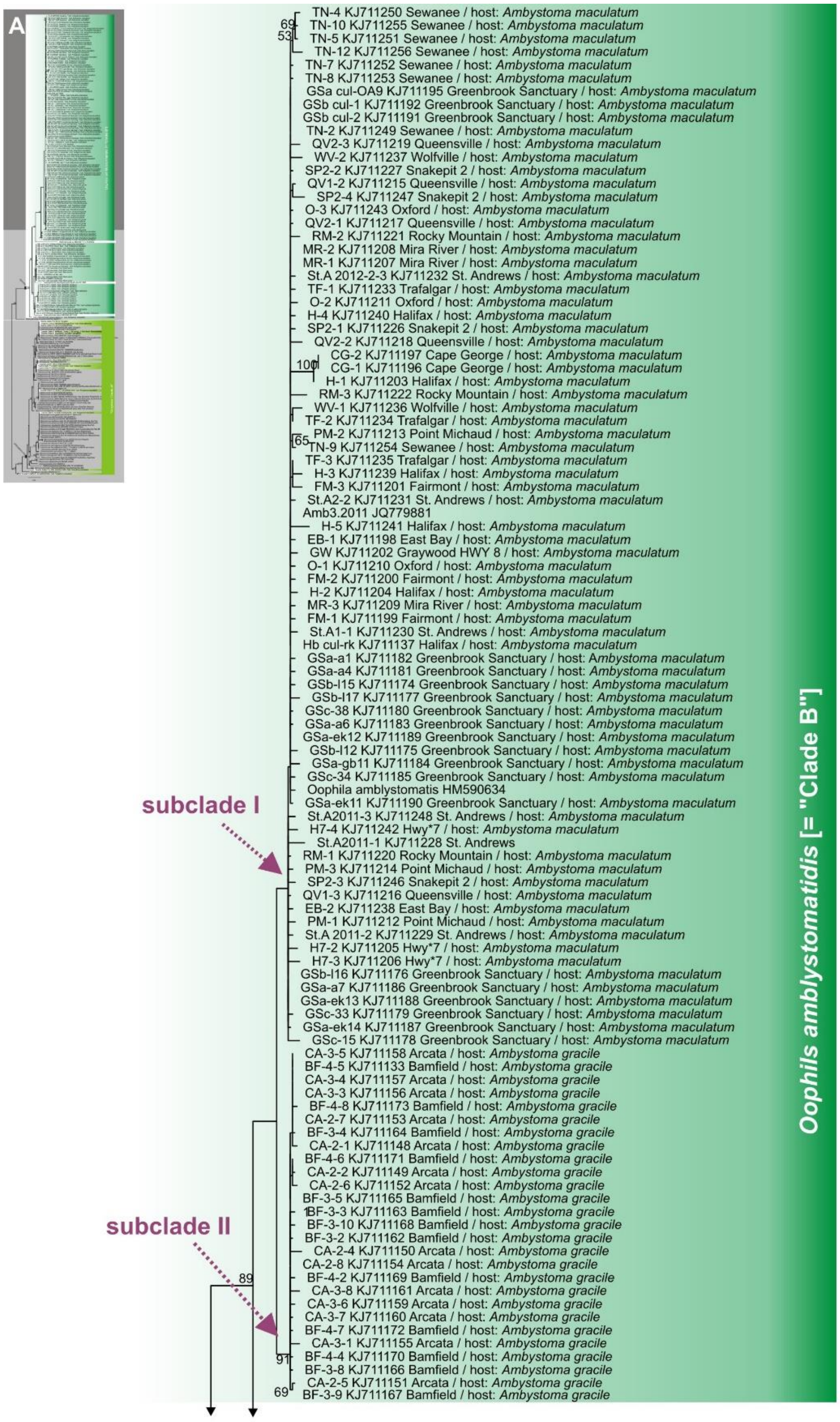

Figure S11. Maximum Likelihood tree inferred from DNA sequences of the 18S rRNA gene (Dataset 3; 1878 bp) comprising all sequences assigned to the *Oophila* clade and the *Chlorococcum* clade in the exploratory analysis of Dataset 1 (Online Resource 4, Fig. S9). Dark green marks the clade of amphibian-associated *Oophila* ("Clade B" according to Nema *et al.* 2019) with subclades according to Kim *et al.* (2014) and Muto *et al.* (2017), light green marks the *Oophila*-like lineages found associated to amphibians within the *Chlorococcum* clade ("Clade A" according to Nema *et al.* 2019). Numbers at nodes show bootstrap values in percent (only shown if >50%, and removed from the shallowest nodes for better graphical representation).

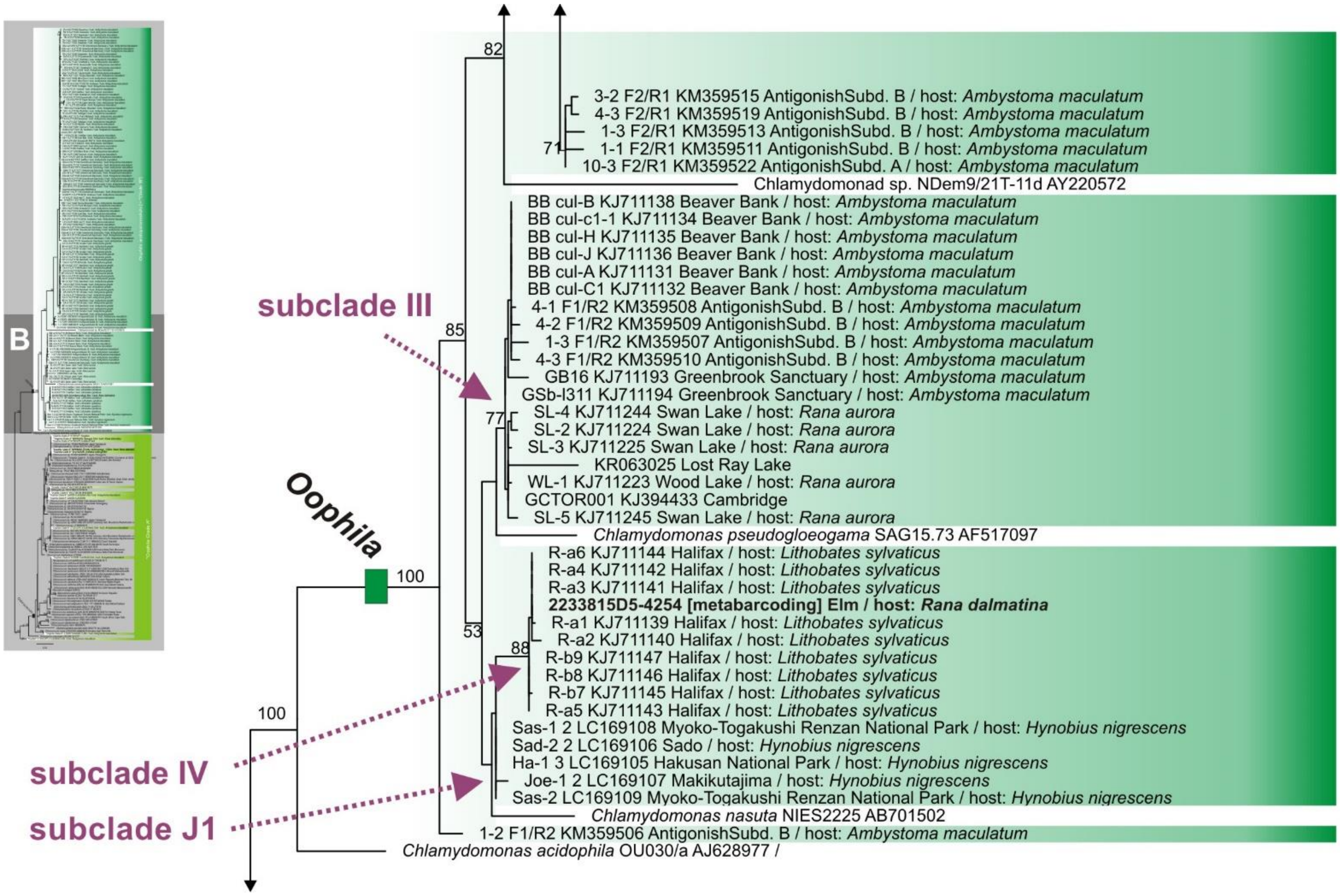

Figure S11. Continued.

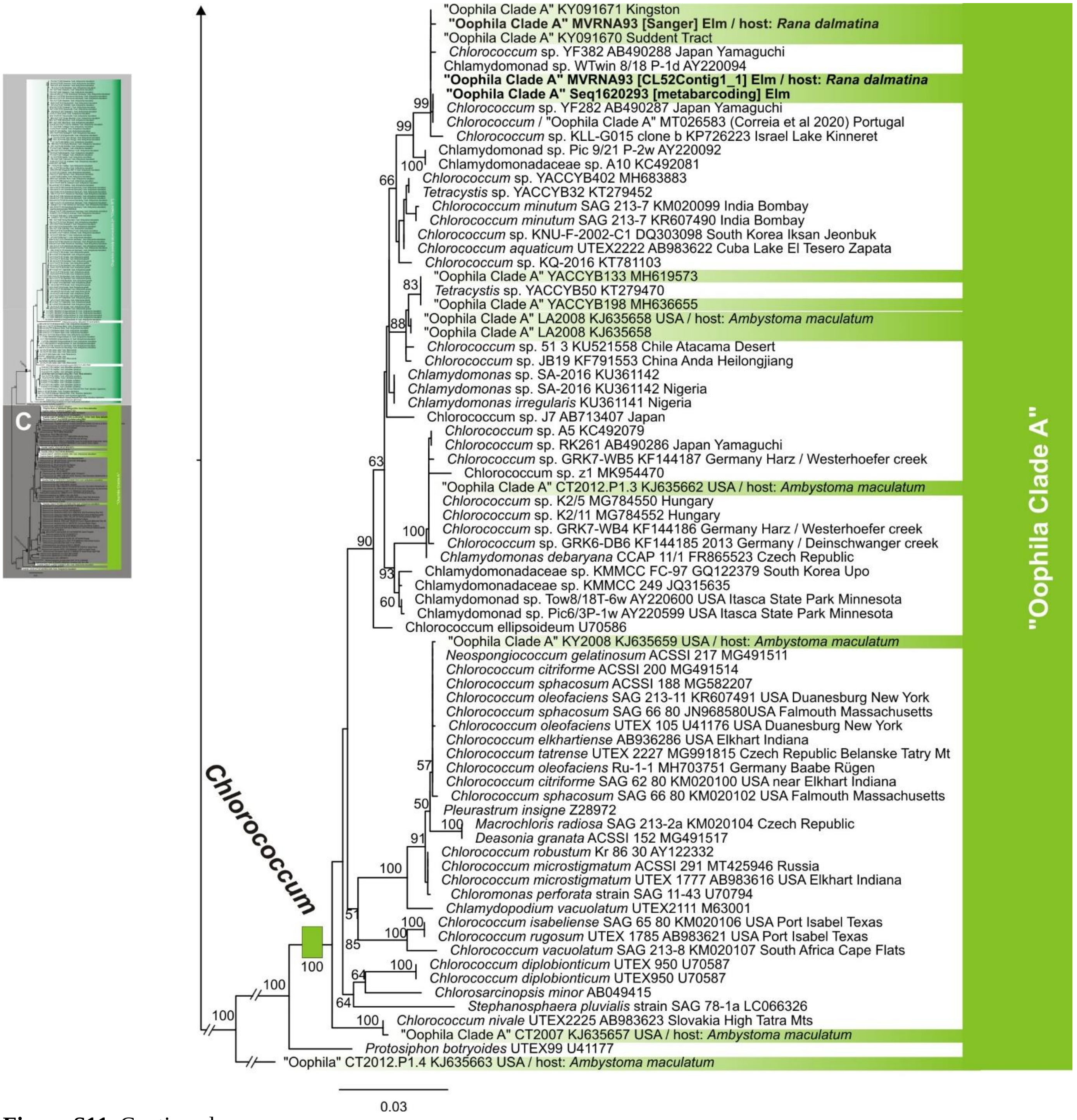

Figure S11. Continued.

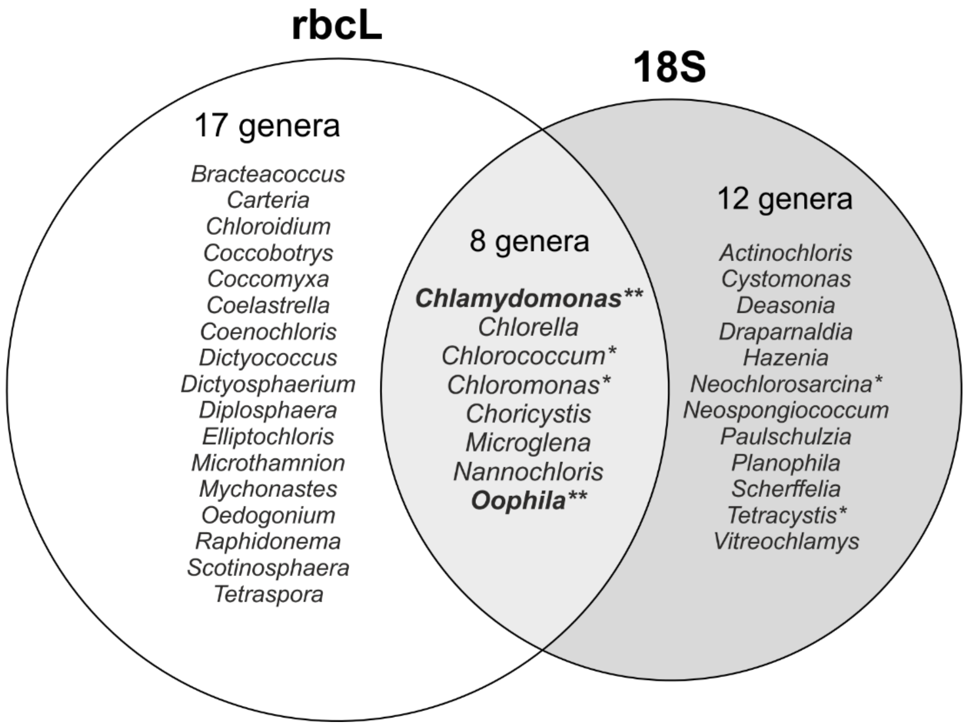

Figure S12. Venn diagram demonstrating the detected clutch-associated green algal genera with rbcL and 18S markers. The respective OTUs in rbcL and 18S data sets were considered to represent noted genera when the blastn identity percentage was ≥ 95% against the noted genus on the diagram. Seventeen clutch-associated genera were unique to rbcL data, 12 genera to 18S data, and 8 genera were found with both markers. An asterisk (*) after the genus name denotes that an OTU within this genus was identified as indicator OTU in 18S data set. Double asterisk (**) denotes that at an OTU within noted genus was identified as indicator OTU in both data sets (rbcL and 18S). Indicator OTUs are outlined in Table S6.

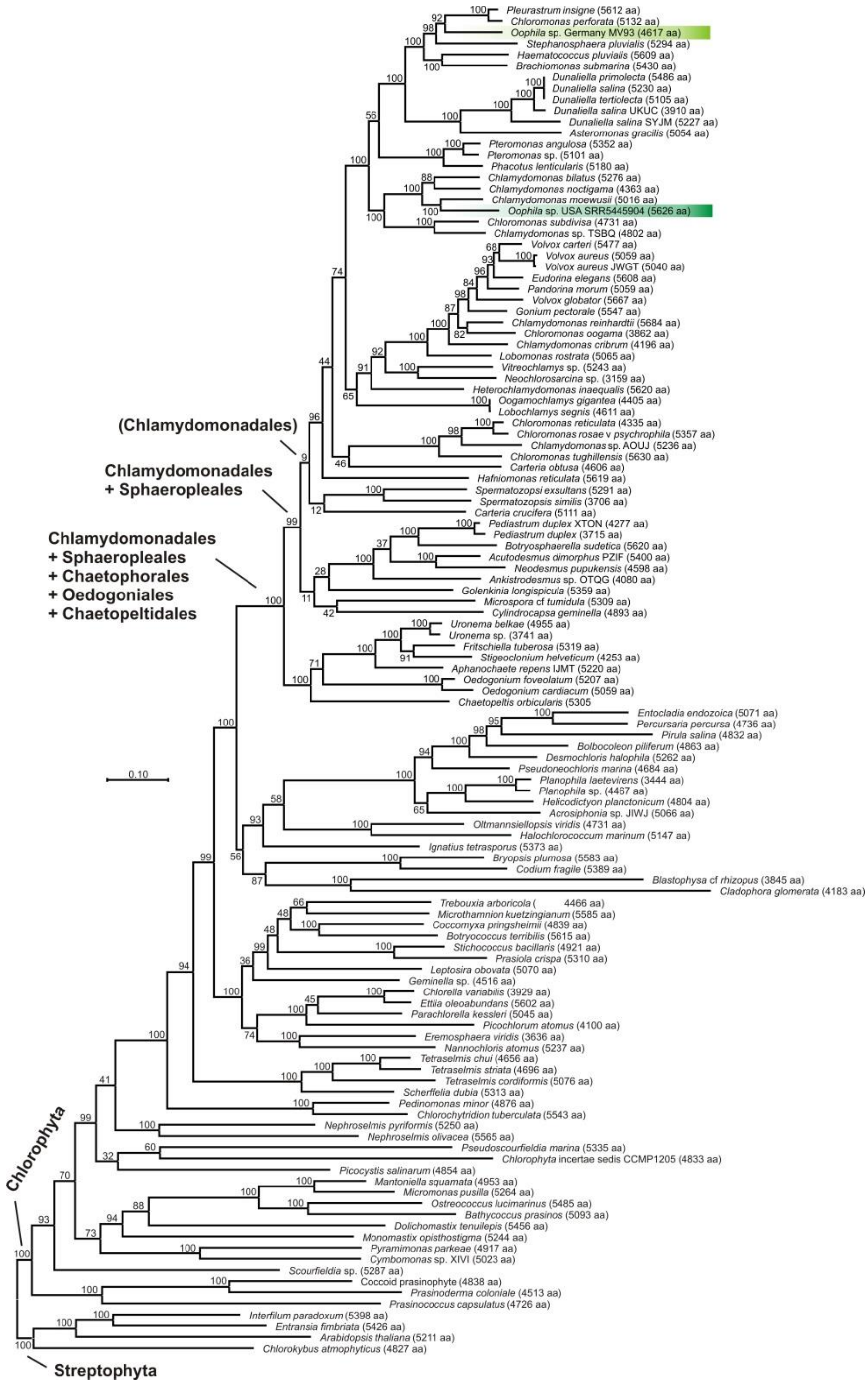

Figure S13. Maximum Likelihood tree inferred from DNA sequences of 18 nuclear protein-coding genes after exclusion of hypervariable regions (Dataset 4; alignment length 4219 amino acids), comprising all Chlorophyta for which sequences were available from the study of (Leebens-Mack et al. 2019), plus sequences extracted from transcriptomes of one *Oophila* strain isolated from a clutch of *Ambystoma maculatum* (dark green; corresponding to "Clade B" of Nema et al. (2019) and of one *Oophila*-like representative of the *Chlorococcum* clade isolated from a clutch of *Rana dalmatina* (light green; corresponding to "Clade A" of Nema et al. (2019). Numbers at nodes show bootstrap values in percent. Four taxa of Streptophyta were used as outgroup.

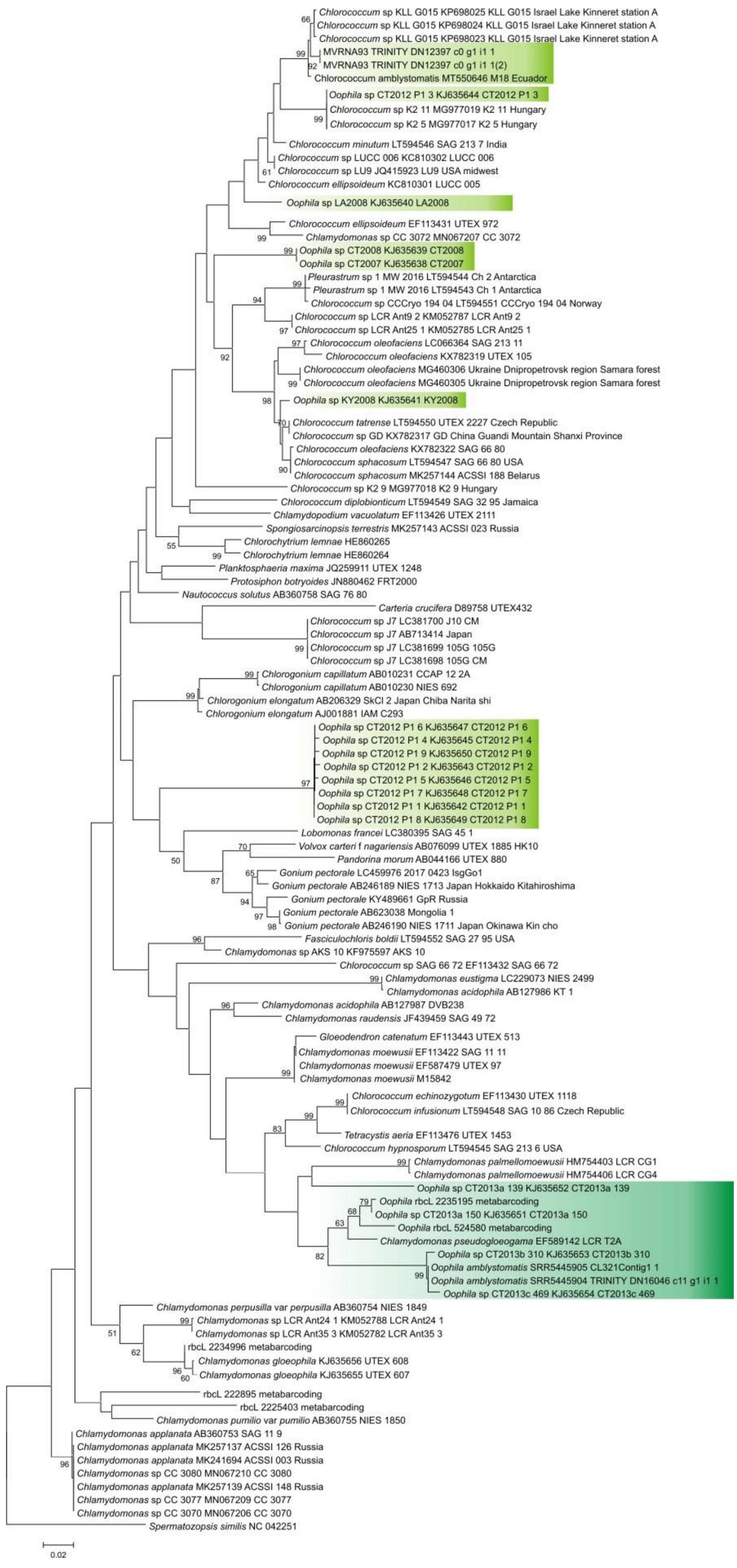

Figure S14. Phylogenetic tree obtained by ML analysis in MEGA (GTR+G model) with 100 bootstrap replicates (values in percent, only shown if >50%), of a 1318 bp alignment of rbcL sequences. Dark green corresponds to "Clade B" of Nema et al. (2019), light green to "Clade A" of Nema et al. (2019).

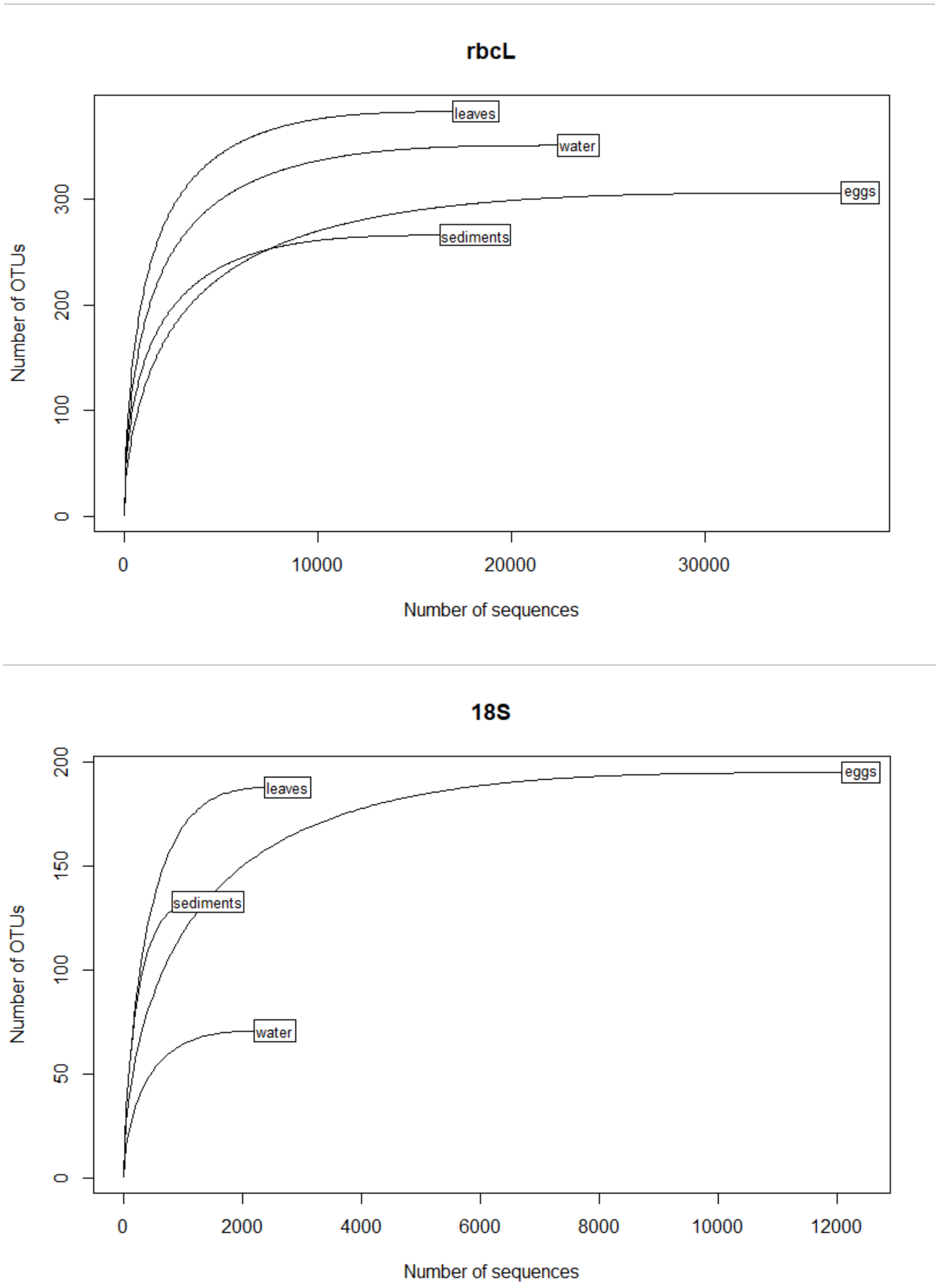

Figure S15. OTU accumulation curves per sampling substrates for rbcL and 18S metabarcoding data. 'Eggs' denote clutches samples. Note that the sequencing depth for leaves, sediments and water samples using 18S marker was relatively low; however, the curves indicate the saturation in OTU accumulations.
